# Men Scare Me More: Gender Differences in Social Fear Conditioning in Virtual Reality

**DOI:** 10.3389/fpsyg.2019.01617

**Published:** 2019-07-18

**Authors:** Jonas Reichenberger, Michael Pfaller, Diana Forster, Jennifer Gerczuk, Youssef Shiban, Andreas Mühlberger

**Affiliations:** ^1^Department of Clinical Psychology and Psychotherapy, Institute of Psychology, University of Regensburg, Regensburg, Germany; ^2^Department of Clinical Psychology, Institute of Psychology, Private University of Applied Science Göttingen, Göttingen, Germany

**Keywords:** social anxiety disorder, social fear conditioning, virtual reality, fear ratings, fear-potentiated startle, avoidance behavior, recognition

## Abstract

Women nearly twice as often develop social anxiety disorder (SAD) compared to men. The reason for this difference is still being debated. The present study investigates gender differences and the effect of male versus female agents in low (LSA) and high socially anxious (HSA) participants regarding the acquisition and extinction of social fear in virtual reality (VR). In a social fear conditioning (SFC) paradigm, 60 participants actively approached several agents, some of which were paired with an aversive unconditioned stimulus (US) consisting of a verbal rejection and spitting simulated by an aversive air blast (CS+), or without an US (CS−). Primary outcome variables were defined for each of the 4 levels of emotional reactions including experience (fear ratings), psychophysiology (fear-potentiated startle), behavior (avoidance), and cognition (recognition task). Secondary outcome variables were personality traits, contingency ratings, heart rate (HR), and skin conductance response (SCR). As hypothesized, fear ratings for CS+ increased significantly during acquisition and the differentiation between CS+ and CS− vanished during extinction. Additionally, women reported higher fear compared to men. Furthermore, a clear difference in the fear-potentiated startle response between male CS+ and CS− at the end of acquisition indicates successful SFC to male agents in both groups. Concerning behavior, results exhibited successful SFC in both groups and a general larger distance to agents in HSA than LSA participants. Furthermore, HSA women maintained a larger distance to male compared to female agents. No such differences were found for HSA men. Regarding recognition, participants responded with higher sensitivity to agent than object stimuli, suggesting a higher ability to distinguish the target from the distractor for social cues, which were on focus during SFC. Regarding the secondary physiological outcome variables, we detected an activation in HR response during acquisition, but there were no differences between stimuli or groups. Moreover, we observed a gender but no CS+/CS− differences in SCR. SFC was successfully induced and extinguished according to the primary outcome variables. VR is an interesting tool to measure emotional learning processes on different outcome levels with enhanced ecological validity. Future research should further investigate social fear learning mechanisms for developing more efficient treatments of SAD.

## Introduction

### Social Anxiety Disorder and Social Fear Conditioning

According to the DSM-5, social anxiety disorder (SAD) is characterized by a persistent fear of one or more social or performance situations in which the person is exposed to unfamiliar people or to possible scrutiny by others, which is often associated with avoidance of social situations (American Psychiatric Association American Psychiatric Association [APA], 2013). With an odds ratio of 1.5–2.2, SAD is nearly twice as prevalent in women than in men (Fehm et al., 2005). Further empirical evidence on gender differences indicates that psychosocial stress affects fear conditioning in men and women differently (Jackson et al., 2006; Zorawski et al., 2006).

Research on fear and anxiety investigates the mechanisms of emotional learning across species with the purpose of improving treatments tackling the severe effects of anxiety disorders in humans. Many researchers use classical fear conditioning, which has been extensively investigated in animals and in healthy and phobic humans. Such learning paradigms use pairings with an initially neutral stimulus (NS) and an aversive unconditioned stimulus changing the NS to a conditioned stimulus (Mertens et al., 2018). However, there are also other conditioning paradigms of aversive learning involving contextual stimuli (Glotzbach-Schoon et al., 2013; Shiban et al., 2013), generalization processes (Lissek and Grillon, 2010; Vervliet and Geens, 2014; Andreatta et al., 2017) or more complex operant or instrumental conditioning, in which behavior is learned by its consequences (Lissek et al., 2005; Shiban et al., 2015; Reichenberger et al., 2017).

In a single-cue conditioning procedure, anxiety patients present enhanced fear conditioning to CS+ compared to healthy humans (Lissek et al., 2005). In a review, Duits et al. (2015) reported increased fear responses to CS− stimuli during fear conditioning and stronger fear responses to CS+ stimuli during fear extinction in anxiety patients compared to healthy controls. In contrast, Ahrens et al. (2015) suggest that patients with SAD exhibit no generally enhanced conditionability but distinguish in discrimination learning of CS+ and CS− (non-reinforced stimuli) and in terms of resistance to extinction compared to healthy controls. Following this, Ahrens et al. (2016) indicate that it is important to differentiate between individual stimuli in order to save resources and to decrease unnecessary fight-or-flight responses or avoidance behavior if there is no threat. Hermann et al. (2002) showed that SAD patients exhibited an enhanced US expectancy during acquisition, especially for the CS−, which indicates an overgeneralization of the conditioned emotional response and contributes to the etiology of SAD. Ahrens et al. (2016) detected that SAD is not characterized by strong overgeneralization but by discrepancies in fear responses to conditioned and generalized threat stimuli.

Due to the use of stimuli with low ecological validity regarding the nature of SAD in most published studies, it is still being debated if socially anxious humans respond more sensitively to socially relevant stimuli. Ahrens et al. (2015) indicated a potentially impaired ability of highly socially anxious participants to discriminate between relevant and irrelevant social stimuli. The social relevance of the US might play a significant role in fear learning, therefore a disorder-relevant US should be used to examine affective learning in SFC paradigms (Lissek et al., 2008). Fear learning outside of the lab hardly exists to simple sensory cues, like geometric, facial or electrical stimuli, but contains more complex stimuli which vary between psychopathological dysfunctions (Ahrens et al., 2016). Furthermore, Ahrens et al. (2016) suggest that fear conditioning in high socially anxious persons or SAD patients can not only be induced by effectively non-social US (e.g., electrical stimuli), but also by social stimuli, like critical facial expressions with verbal feedback (Lissek et al., 2008), isolated verbal comments (Ahrens et al., 2015) or spitting with verbal rejection (Reichenberger et al., 2017).

Many researchers demand an expansion of the human fear conditioning paradigm by comprising behavioral tendencies as a significant index of fear and focusing more on uncertain than strong fear learning situations, such as contained in single-cue or differential fear conditioning (Lissek et al., 2006; Glotzbach et al., 2012; Beckers et al., 2013). Following this, it is important to construct situations with an amount of complexity and uncertainty. Furthermore, individual factors, e.g., personality traits and dispositions, should be emphasized more in future research as they are predictive for the development of anxiety disorders (Beckers et al., 2013).

The conceptual−theoretical model for SAD (Rapee and Heimberg, 1997) suggest a coupling of a cue (e.g., facial expression, verbal rejection) or situation (e.g., a social situation), emotional and physiological reactions (e.g., fear-potentiated startle reaction), behavior (e.g., approach-avoidance behavior), and cognitions (e.g., threat thoughts). In the following paragraph, each aspect will be explained in more detail in light of social fear conditioning.

### Social Situations

According to core fear in SAD (see DSM-5 definition of SAD, American Psychiatric Association American Psychiatric Association [APA], 2013) empirical research should investigate social or performance situations in which the person is exposed to possible scrutiny by others. However, *in vivo* studies make a great effort in planning and organizing a social situation, there is less control over the situation or a counterpart (e.g., social skills training), as well as a higher inhibition threshold for patients (e.g., *in vivo* exposure therapy). In VR participants have the ability to interact with diverse stimuli (e.g., human agents) and their environment similar to an *in vivo* social situation. Hence, SFC in VR offers a great benefit to simulate social interactions with a high controllability and thereby to enhance ecological validity compared to traditional cue-conditioning. VR conveys the feeling of being present in the virtual situation by providing immersive sensory perceptions and allowing interactions with the virtual environment. The feeling of presence might be a crucial factor to investigate social interactions and social fear as it is relevant in real life situations (Diemer et al., 2015). Our SFC paradigm offers basic social interaction opportunities between the participant and the agent (e.g., eye contact, self-regulated movement of the avatar and movement toward the agent). In addition, the facial expressions of the agents are paired with a verbal rejection in order to enhance the US (see Reichenberger et al., 2017). Besides many further advantages with VR, e.g., realistic, standardized environment in an economic and easily administrable manner, the results of conditioning processes in VR are hugely satisfying (Huff et al., 2010).

### Emotional and Physiological Reactions

According to Lang’s fear response system (Lang, 1968), following outcome measures of conditioned fear are commonly used in fear conditioning: subjective, physiological, and behavioral fear responses (Mertens et al., 2018). In VR we can directly measure the reactions of the participants to the stimuli via subjective verbal ratings (e.g., fear and contingency ratings), physiological (e.g., fear-potentiated startle, electrodermal activity or electrocardiographic) and behavioral data (Mühlberger et al., 2006, 2007; Powers and Emmelkamp, 2008; Shiban et al., 2013; Kinateder et al., 2015). Physiological outcome measures are commonly applied and offer the advantage of being less conscious and less vulnerable to bias in comparison to subjective reports (Lonsdorf et al., 2017). For example, fear-potentiated startle is a less cognitive index of fear than verbal ratings, but also than skin conductance responses (SCRs) (Lipp et al., 2003; Mallan et al., 2009; Beckers et al., 2013).

### Behavior

According to Beckers et al. (2013) another important outcome measure is the behavioral fear response in anxiety research. Commonly, an adaptive behavioral strategy is to avoid threatening or unpleasant events. Nevertheless, excessive threat-avoidance is a central diagnostic feature and a known risk factor for the acquisition and maintenance of anxiety disorders (Dymond et al., 2012). Socially anxious humans are more likely to avoid new social situations if aversive consequences occur (Mineka and Oehlberg, 2008). However, individuals with SAD do not always avoid social situations, but sometimes endure them with high levels of distress (Hofmann et al., 2009). Mertens et al. (2018) demonstrate that measuring behavioral fear responses typically involve the physical distance to and the degree of interaction with the CSs using behavioral approach tests (e.g., time for approaching, pressing a button to remove the stimulus or to avoid the US, attentional bias). Besides gender differences in the prevalence of SAD, the gender of the counterpart might also be an influencing factor in the behavioral fear response. In their study on distance in social interactions in VR, Iachini et al. (2014) showed that participants of both genders approach females more closely than male agents. Interpersonal space is defined as a safety space contributing to protect the body from an external threat (Iachini et al., 2014, 2016; Cartaud et al., 2018). Social anxiety is characterized by a prevalence of enlarged interpersonal space for pleasant social interaction (Cartaud et al., 2018). Moreover, Åhs et al. (2015) showed that interpersonal defensive boundaries increase with aversive learning.

### Cognitive Processing

Cognitive models of SAD suggest self-perception as an important maintaining factor of the disorder (Clark and Wells, 1995). Individuals with SAD are characterized by negative cognitions like a negative view of themselves and their social skills in social interactions in detail. A further frequently occurring phenomenon is post-event rumination, in which individuals recall the interaction as being more negative than it actually was (Hofmann, 2007). Besides negative cognitions, it raises the question how deeply the social interaction is processed and stored in memory. The signal detection theory (SDT) offers an empirical examination of memory processing and performance in SAD. With the use of the SDT, statistical analysis allows for the differentiation between discrimination ability (sensitivity) and particular response criteria (Velden, 1982; Sawchuk et al., 2002).

Empirical findings of anxiety assume that an information processing bias exists in the detection, discrimination, and response to stimuli that indicate threat and safety. Thus, anxious persons should show different patterns of sensitivity and response criteria toward threat stimuli (Foa and Kozak, 1986; Sawchuk et al., 2002). Pérez-López and Woody (2001) examined recognition memory for reassuring and threatening facial expressions in individuals with SAD and healthy controls. Individuals with SAD were less sensitive to recognizing previously seen stimuli compared to controls but showed no memory bias toward threatening facial expressions. Further empirical studies reported that socially phobic participants are more likely to interpret ambiguous social interactions as negative and are better at remembering negative or critical rather than neutral or happy facial expressions, whereas healthy controls show no such differences (Lundh and Öst, 1996; Gilboa-Schechtman et al., 1997).

The processing of emotional facial expressions has usually been investigated using a series of isolated, de-contextualized, static photographs of humans posing with facial expressions that enhance the dissimilarity among categories (Wieser and Brosch, 2012). However, human face perception and evaluation are always affected by emotional and non-emotional aspects of context features and are regulated by individual traits such as social anxiety (Schwarz et al., 2013). Therefore, the question arises whether SFC leads to improved memory representations for conditioned stimuli.

### SFC Paradigm

In previous SFC studies, we could successfully induce and extinguish social fear, which emphasizes the role of conditioning in social fear learning (Shiban et al., 2015; Reichenberger et al., 2017). The current study investigates social fear conditioning in VR in a human sample using our SFC setting, which consisted of a CS habituation, acquisition, and extinction phase similar to both our preliminary studies. Furthermore, we maximized the difference between participants by analyzing low (LSA) and high (HSA) socially anxious participants as well as women and men in an equal ratio. Furthermore, we implemented female and male virtual agents into our SFC paradigm to investigate whether there is dissimilarity between learning and unlearning of social fear in women and men and whether there is an influence of the gender of social stimuli (female vs. male agents). In this way, we will investigate how female or male participants behave toward female or male virtual agents to assess affective learning processes in social anxiety.

In addition to that, we added an unforeseen recognition memory task following the SFC paradigm in VR to investigate recognition processes in LSA and HSA participants. We presented stimuli and distractors of the previously seen female and male agents as well as items like tables, chairs and cupboards in the VR environment during the SFC paradigm.

A further feature of our paradigm is the high belongingness between the CS and the US, which is a possible advantage in recruiting clinical samples (Hamm et al., 1989; Lissek et al., 2008). Moreover, the paradigm involves a degree of complexity and uncertainty. Another advantage is that we use disorder-relevant stimuli to improve the ecological validity of the SFC paradigm.

### Hypotheses

The present study investigates whether gender differences occur in social fear conditioning with female and male agents. We tested low and high socially anxious female and male participants in our SFC paradigm in VR with an enhanced ecological validity. Participants actively approached virtual female and male agents using a joystick. According to the conceptual-theoretical model for SAD (Rapee and Heimberg, 1997), we aimed at covering the subjective, physiological, behavioral, and cognitional effects of the acquisition and extinction of social fear. Therefore, we assessed each level of emotional responses on primary outcome (fear ratings, fear-potentiated startle, avoidance, recognition memory performance) and additional secondary outcome variables (personality traits and dispositions, contingency ratings, SCR, HR response). Based on the aforementioned findings, for the acquisition phase we hypothesized that (1) fear ratings for CS+ would increase compared to CS− for pre to post acquisition measurement. Concerning the contingency ratings, we expected increased US expectancy for the CS− post acquisition in HSA compared to LSA. Furthermore, the amplitude of the fear-potentiated startle, the SCR, and the HR response approaching the CS+ and the avoidance behavior during the approach toward CS+ were expected to be higher compared to CS−. After the extinction phase, (2) fear and contingency ratings of the CS+ were supposed to return to baseline levels along with physiological and behavioral outcome variables. (3) According to Duits et al. (2015), we expected increased fear responses to CS− stimuli during fear acquisition and stronger fear responses to CS+ stimuli during fear extinction among HSA compared to LSA participants. (4) Acquisition and resistance to extinction were expected to be higher for women than for men. In addition, we expected to find higher fear responses when approaching male agents in comparison to female agents. (5) We hypothesized that for HSA, recognition memory for CS+ agents would be greater than for CS− and NS agents compared to LSA participants. Finally, (6) we hypothesized that HSA would show a generalization effect for the NS during the extinction phase compared to LSA participants.

## Materials and Methods

### Participants

Seventy healthy volunteers were recruited through advertisements and a screening in first semester events at the University of Regensburg. Hundred and eighty students took part in the screening and questionnaires were used to assess demographic data, exclusion criteria, and different personality traits and dispositions as well as social anxiety using the Social Phobia Inventory (SPIN; Connor et al., 2000; German version: Stangier and Steffens, 2002). The allocation to the low (LSA) and high socially anxious (HSA) group was based on the SPIN cut-off value of 19 stated in the screening. According to Connor et al. (2000), a SPIN score of 19 distinguishes patients with SAD from healthy subjects with a diagnostic accuracy of 79%.

Exclusion criteria being below 18 or above 55 years of age, a current diagnosis of a mental or neurological disorder (excluding SAD), current psychotherapeutic, psychiatric or neurological treatment, history of psychotropic drug use, and pregnancy or lactation, as well as participation in a former social fear conditioning study at the University of Regensburg. These criteria were assessed via a questionnaire after written informed consent had been obtained.

As 10 participants were excluded due to cybersickness symptoms (*n* = 4) or technical error during data acquisition (*n* = 6), the study comprised a total of 60 participants (31 participants in the LSA group: 51.6% female, aged between 18 and 43; and 29 participants in the HSA group: 51.7% female, aged between 19 and 33). The allocation of the participants into a low and high socially anxious group can be regarded as successful, since HSA show significantly higher values with large effect sizes in the SPIN, in the Social Interaction Anxiety Scale (SIAS; Mattick and Clarke, 1998; German version: Stangier et al., 1999) and in the Anxiety Sensitivity Index-3 (ASI; Taylor et al., 2007; German version: Kemper et al., 2009) compared to the LSA group (see Table 1).

**TABLE 1:** Significant results of the ANOVA for fear ratings of the acquisition and extinction phase.

**Effect**	***df***	***F***	**$\eta_{p}^{2}$**	***p***
**Acquisition**				
Total				
Phase	1, 56	22.1	0.28	< 0.001
Stimulus	1, 56	33.2	0.37	< 0.001
Gender	1, 56	4.43	0.07	0.040
Agent ×Social Anxiety	1, 56	4.26	0.07	0.044
Stimulus × Gender	1, 56	4.48	0.07	0.039
Phase × Stimulus	1, 56	33.3	0.37	< 0.001
Phase × Stimulus × Gender	1, 56	4.47	0.07	0.039
Phase × Stimulus × Gender × Social Anxiety	1, 56	4.33	0.07	0.042
LSA				
Phase	1, 29	12.9	0.31	< 0.001
PStimulus	1, 29	17.4	0.38	< 0.001
PAgent	1, 29	4.78	0.14	0.037
PPhase × Stimulus	1, 29	14.4	0.33	< 0.001
PPhase × Gender	1, 29	4.55	0.14	0.041
HSA				
PPhase	1, 27	10.1	0.27	0.004
PStimulus	1, 27	15.9	0.37	< 0.001
PPhase × Stimulus	1, 27	18.6	0.41	< 0.001
PPhase × Stimulus × Gender	1, 27	7.31	0.21	0.012
HSA women				
PPhase	1, 14	5.86	0.30	0.030
PStimulus	1, 14	11.0	0.44	0.005
PPhase × Stimulus	1, 14	16.0	0.53	< 0.001
HSA men				
PPhase	1, 13	5.25	0.29	0.039
PStimulus	1, 13	7.21	0.36	0.019
**Extinction**				
Total				
PPhase	1, 56	22.1	0.28	< 0.001
PStimulus	1, 56	37.9	0.40	< 0.001
PAgent	1, 56	5.67	0.09	0.021
PGender	1, 56	4.50	0.07	0.038
PStimulus × Gender	1, 56	5.55	0.09	0.022
PPhase × Gender	1, 56	5.11	0.08	0.028
PPhase × Stimulus	1, 56	21.1	0.27	< 0.001
PPhase × Stimulus × Agent	1, 56	4.02	0.07	0.050
Female agent				
PPhase	1, 59	26.4	0.31	< 0.001
PStimulus	1, 59	30.2	0.34	< 0.001
PPhase × Stimulus	1, 59	27.1	0.32	< 0.001
Male agent				
PPhase	1, 59	28.3	0.32	< 0.001
PStimulus	1, 59	28.8	0.33	< 0.001
PPhase × Stimulus	1, 59	9.96	0.14	0.003

**df*, degrees of freedom;$\eta_{p}^{2}$, effect size; *Phase*, pre- vs. post-acquisition for acquisition and post-acquisition vs. post extinction for extinction; *Stimulus*, CS+ vs. CS−; *Agent*, female vs. male agent; *Gender*, women vs. men; *Social Anxiety*, LSA vs. HSA.*

All participants had unimpaired or corrected vision or hearing. All of the volunteers were students at the University of Regensburg and were offered credit points as compensation for their participation. The Ethics Committee of the University of Regensburg approved the study.

### Apparatus

**FIGURE 1:**
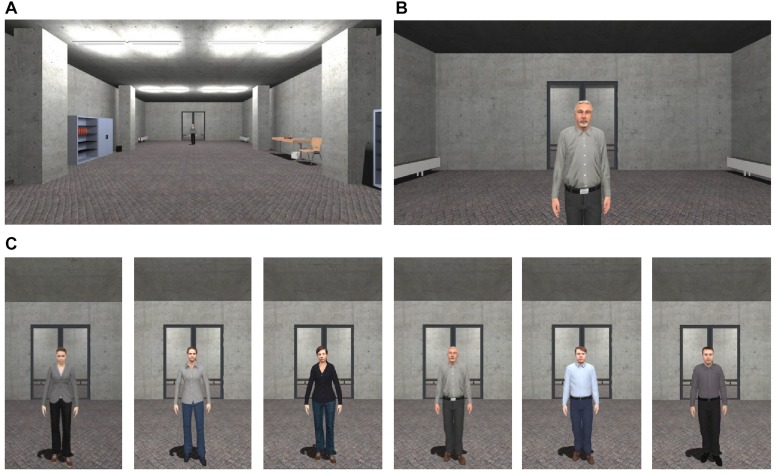
Virtual environment. **(A)** Starting point in the room, in which learning phases (CS habituation, acquisition, and extinction), ratings, and behavioral avoidance task took place. **(B)** End point for approaching the agent. **(C)** Social stimuli (agents) used for social fear conditioning.

The VR environment consisted of one room, in which all three phases (CS habituation, acquisition, and extinction), the subjective ratings and the behavioral avoidance tests (BAT) took place. In each phase, the participant was positioned at one end of the room and could see each female or male agent at the opposite end of the room. The agents gazed dynamically at the participant and moved their head and upper body slightly (see Figure 1). In 75% of the conditioning trials, an aversive consequence followed as soon as the participants reached a specific distance to the agents (30 cm) and navigation stopped. Aversive consequences consisted of an air blast to the right side of the participant’s neck (5 bar, 10 ms) accompanied by a sound of spitting followed by a verbal rejection. A compressed tank of air, which was regulated via a magnetic valve system, channeled the air blast through a tube that was fixed to the participant’s torso. In addition, when the participant approached the agent, a startle sound was administered in 75% of all trials in all phases.

The VR was presented to participants via the HTC VIVE head-mounted display (HMD; HTC Corporation, Taoyuan, Taiwan) and was generated via the Steam Source engine (Valve Corporation, Bellevue, WA, United States). The presented VR environment was controlled by CyberSession Research 5.6 (VTplus GmbH, Würzburg, Germany). The participant’s head position was monitored via the HTC VIVE lighthouse tracking system, which creates a 360-degree virtual space with up to 15 × 15 foot radius (HTC Corporation, Taoyuan, Taiwan). This tracking data is transmitted in real time to the CyberSession software using the Valve OpenVR^1^, which adjusted the participant’s field of view according to the tracked head position. Sounds were presented over headphones (Sennheiser HD-215, Sennheiser electronic GmbH, Germany). Participants used a joystick (Logitech Extreme 3D Pro Joystick, Logitech GmbH, Germany) mounted on a stand to move in the VR environment. Physiological data were monitored, digitally amplified (V-Amp, Brain Products GmbH, Germany) and recorded (Brain Vision Recorder software, Version 1.20, Brain Products GmbH, Germany).

### Measures

Participants filled out a demographic questionnaire (age, sex, education, current occupation, and exclusion criteria) and the following questionnaires.

The SPIN was used to assess social anxiety and consists of 17 items that assess fear, avoidance, and physiological symptoms of SAD in the previous week. Answers are given on a five-point Likert scale (from 0 = “not at all” to 4 = “extremely”). The German version of the SPIN was evaluated by Sosic et al. (2008). Internal consistency was excellent for a representative sample of 2043 Germans (Cronbach’s α = 0.95). Convergent and divergent validity are satisfactory. Furthermore, the German version of the SPIN is a sensitive and specific measure for SAD as it distinguishes successfully between SAD and other psychiatric disorders (Sosic et al., 2008).

The SIAS assesses anxiety in social interactional situations with 20 items on a five-point Likert scale from 0 = “not at all” to 4 = “very strong.” The internal consistency of the German version can be classified as extremely reliable with a Cronbach’s α = 0.94 (Heinrichs et al., 2002). Construct and convergent validity were rated as good and reliability as good to excellent (Heimberg and Turk, 2002).

The ASI-3 was used to assess participants’ fear of physiological arousal related sensations. The questionnaire captures three dimensions of anxiety sensitivity (physical, social, and cognitive concerns) with 18 items on a five-point Likert scale (from 0 = “do not agree” to 4 = “completely agree”). The reliability depends on the sample and ranges from acceptable to excellent (Cronbach’s α = 0.75 – 0.92). Factorial and construct validity are satisfactory (Kemper et al., 2009).

The Dominance scale of the German Personality Research Form (PRF-D; Stumpf et al., 1985) was employed to quantify the characteristics of dominant behaviors. It investigates dominant personality traits via preferences and behaviors with 16 items on a dichotomous scale (“right” vs. “false”). In a sample of 1086 participants, the PRF-D achieved a good internal consistency (Cronbach’s α = 0.82). Furthermore, retest-reliability (0.84) as well as convergent and discriminant validity are high (Stumpf et al., 1985).

The Submissive Behavior Scale (SBS; Allan and Gilbert, 1997), which was translated into German for this study, assesses the frequency of submissive behavior with 16 items rated on a five-point Likert scale from 0 = “never” to 4 = “always.” Internal consistency proved to be high both in a student, Cronbach’sα = 0.82, and a clinical sample, Cronbach’s α = 0.85 (Allan and Gilbert, 1997).

The subtest Ego threat of the German version of the Mainz Coping Inventory (MCI; Krohne et al., 2000; German version: Krohne and Egloff, 1999) captures two coping strategies, Vigilance (VIG) and Cognitive Avoidance (CAV), with 10 items respectively with regard to four stressful situations (public speech, examination, job application, mistake on the job), which are answered by agreement or disagreement. Reliabilities of the CAV (Cronbach’s α = 0.76) and VIG (Cronbach’s α = 0.80) are satisfactory (Krohne and Egloff, 1999).

The Simulator Sickness Questionnaire (SSQ; Kennedy et al., 1993) was used to assess symptoms of cybersickness. The questionnaire consists of 16 items and uses a four-point Likert scale (from 0 = “not at all” to 3 = “heavy”) to measure potential side effects of VR (e.g., dizziness symptoms). The Igroup Presence Questionnaire (IPQ; Schubert, 2003) consists of 14 items and captures the experience of presence in the virtual environment on a seven-point Likert scale.

In order to measure the experienced fear (“On a scale from 0 to 100, how intense is your fear during the presence of this person?”) and contingency (“On a scale from 0 to 100, how likely is an unpleasant stimulus during the presence of this person?”) regarding the CS, ratings were assessed verbally on a range from 0 (very low fear/very unlikely) to 100 (very high fear/very likely) during the presentations of the agents in the rating phase following each of the three phases.

Besides these self-reported measures, physiological and behavioral data were collected. To record the electromyography of the musculus orbicularis oculi as a measure of a fear-potentiated startle, four surface electrodes (Ag/AgCl, Ø D 8 mm) were affixed under the right eye of the participant and on the mastoid bones as reference and ground electrodes. Two additional surface electrodes (Ag/AgCl, Ø D 8 mm) were placed on the base of the thumb on the radial side of the palm of the non-dominant hand in order to record the SCR. Two adhesive pre-gelled surface electrodes (Ag/AgCl, Ø = 40 mm) were attached to the middle of the upper chest and on the rib tip of the left half of the body to record the electrocardiography. Avoidance behavior was measured as the minimal distance (in m) to the agent while passing it during the BAT. Recognition processing data were measured as sensitivity *d’* and response criteria β (Velden, 1982).

### Procedure

**FIGURE 2:**
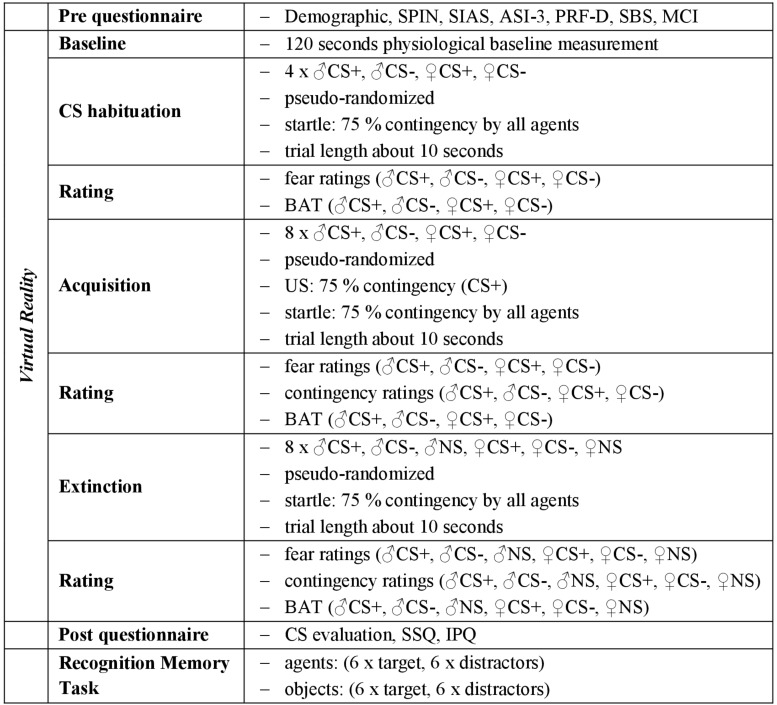
Experimental procedure. The experimental procedure took place as described above. As unconditioned stimulus (US) an air blast combined with virtual spitting and rejection was applied. CS+, agent paired with aversive US; CS–, agent without aversive US; NS, agent without aversive US and only appearing during the extinction phase. SPIN, Social Phobia Inventory; SIAS, Social Interaction Anxiety Scale; ASI-3, Anxiety Sensitivity Index; PRF-D, German Personality Research Form – Dominance Scale; SBS, Submissive Behavior Scale; MCI, Mainz Coping Inventory; SSQ, Simulator Sickness Questionnaire; IPQ, Igroup Presence Questionnaire.

The experiment consisted of filling out questionnaires, the social fear conditioning paradigm in VR (with a CS habituation, acquisition, and extinction phase), further questionnaires after the session in VR and a final recognition memory task (see Figure 2).

At the beginning, participants were briefed in written form and the informed consent was signed. After filling out the questionnaires (demographic, SPIN, SIAS, ASI-3, PRF-D, SBS, MCI), participants were prepared for the VR part of the experiment. The electrodes, the air blast device, the HMD and the headphones were adjusted. During the experiment, participants received recorded instructions, which were delivered via the headphones.

At first, participants relaxed for 2 min in VR (gray screen) for a physiological baseline-measure. Subsequent to nine presentations of the startle noise for habituation of the fear-potentiated startle reaction, participants were given a short task to explore the virtual environment to learn the navigation in VR. Following this, participants received the recorded instruction: “You will now meet several human beings. Please try to move directly toward the persons until they are right in front of you.” Participants had to approach the agents actively using the joystick and as soon as they reached a specific distance to the agents (the equivalent of about 30 cm in the real world), lights faded out and the next agent was presented at the opposite end of the room. Each trial lasted about 10 s (depending on how fast participants approached the agents). Theoretically, participants could move laterally, diagonally or away from the agent, however, we observed no such behavior.

The CS habituation phase consisted of four blocks. One block consisted of one presentation of each female and male agent (CS+, CS−), resulting in a total of 16 presentations. Which agent was presented as CS+ or CS− was balanced across participants. A startle noise (white noise: 103 dB, 10 ms) was presented at two-thirds of the approach path to the agent with a contingency of 75% in each trial. The unconditioned stimulus (US) was not presented yet.

Afterward, the first rating and BAT took place. Participants approached each of the two female and male agents and as soon as they reached the previously specified distance to the agents, lights faded out and the participants were asked to verbally rate their subjective fear. Following the respective ratings, lights faded in again and the participants were instructed to pass the agent and to leave the room through the glass door located behind the agent (“Now go through the glass door behind the person and leave the room”).

The acquisition phase was conducted in eight blocks. One block consisted of one presentation of each conditioned stimuli (female and male CS+) with and without the US (female and male CS−), resulting in a total of 32 presentations. The US was an air blast combined with virtual spitting and the negative rejection “Get lost!”. The facial expressions of the agents are adjusted to the spitting and verbal rejection. The CS–US contingency was set at 75% and the startle noise was also presented with a contingency of 75% in this phase.

After the conditioning phase, the second rating took place. Participants approached each of the two female and male agents and rated their subjective fear and the contingency of aversive events. Subsequently, the BAT took place again as described above. Afterward, there was a 5 min break, during which participants took off the HMD, sat down, and had the opportunity to close their eyes and relax.

The extinction phase consisted of eight blocks designed in exactly the same way as those in the acquisition phase, except for the absence of the US and the appearance of the NS agent of both genders. The total number of trials was 48 in this phase. Just like in the previous phases, the startle noise was presented with a contingency of 75%.

After the extinction phase, the last rating took place. Participants rated their subjective fear and the contingency of aversive events and the third BAT took place for each female and male agent (CS+, CS−, NS).

**FIGURE 3:**
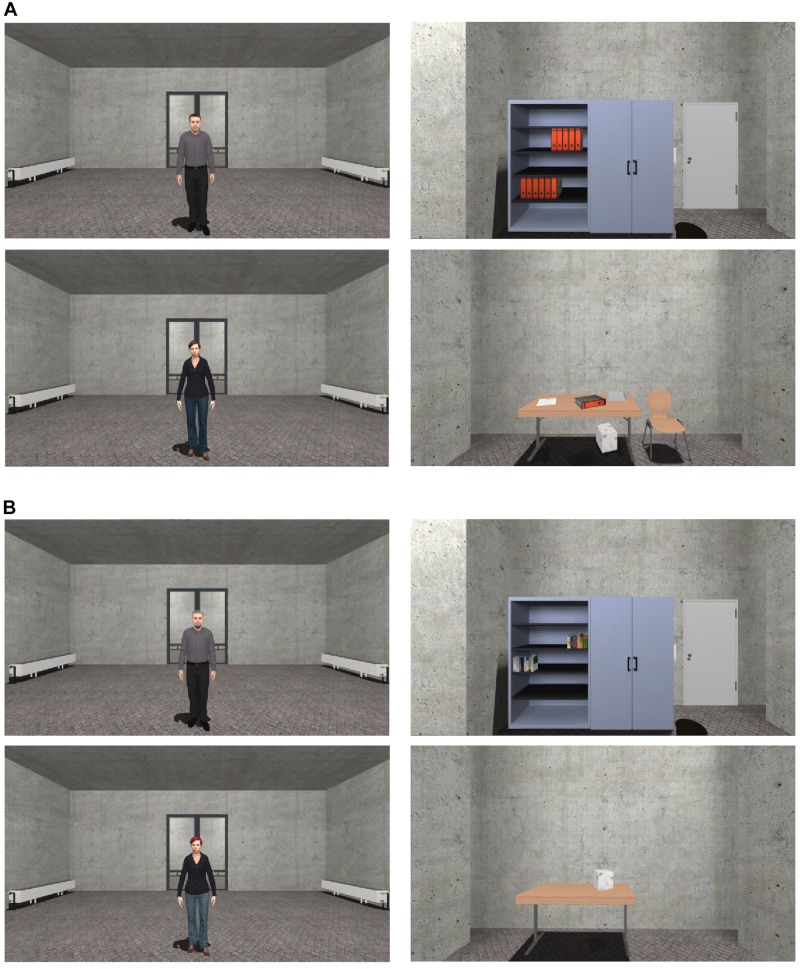
Stimuli of the recognition memory task. Stimuli target **(A)** and distractors **(B)** of the agents and objects were presented. The images were shown on a computer screen in the same size (1600 × 900). For interpretation of the references to color in this figure, the reader is referred to the web version of this article.

After the VR experiment and filling out the last questionnaires (short evaluation of the US, SSQ, IPQ), a surprise recognition memory task was conducted. Twenty-four images of virtual agents and objects (e.g., a chair, table, bookshelf, black board) were shown on a computer screen (1600 × 900) and the participants had to decide whether the presented 12 agents and 12 objects appeared in the virtual environment or not (see Figure 3). Six of these agents and six objects were shown in VR (stimulus) and six were modified (distractor). Participants used a keyboard to indicate if the agent or object was in the experiment (“y” = “yes”) or if it was a modified representation and not presented in the experiment (“n” = “no”). The number of correct answers in the task was recorded. The total duration of the experiment was about 90 min.

### Data Reduction and Statistical Analyses

Physiological data were preprocessed with Brain Vision Analyzer 2.0 software (BVA; Brain Products GmbH, Munich, Germany), the behavioral data were preprocessed with MATLAB 9.5 (MathWorks Inc., Germany) and further analyses were performed in SPSS 22.0 (IBM Corp., Armonk, NY, United States).

For the fear-potentiated startle, first, differences between the two electromyography electrodes were computed (see Blumenthal et al., 2005). Then, a 250 Hz high cut-off filter, a 30 Hz low cut-off filter, and a 50 Hz notch filter were applied, the data were rectified, and a moving average (50 ms) was calculated. For each fear-potentiated startle, a baseline correction was conducted using the mean value of the 50 ms before each startle tone as baseline. Next, peaks between 20 and 120 ms were marked automatically, controlled manually and corrected if necessary. Finally, *T*-values for the startle magnitude were calculated.

For the analysis of the SCR, the difference between the two electrodes was computed and a 1 Hz high cut-off filter was applied. For each SCR, a baseline correction was conducted using the mean value of 500 ms before each presentation of the stimulus as a baseline. SCR was registered as the peak response in the 0.5 – 6 s following stimulus presentation. Peaks were marked automatically, manually controlled and corrected if necessary (see Pineles et al., 2009; Shiban et al., 2016). SCR values < 0.01 μs were defined as non-responses and were set on zero (Lonsdorf et al., 2017). Furthermore, participants with more than 75% non-responses in all trials were excluded from the analysis (Lonsdorf et al., 2017). We had to exclude 8 female and 3 male participants for the SCR analysis.

For heart rate (HR), the difference between the ECG electrodes were computed, a 1.59 Hz (12 dB) high cut-off filter, a 30 Hz (12 dB) low cut-off filter, and a 50 Hz notch filter were administered. Then R-spikes were automatically detected and counted by an algorithm in BVA software, manually controlled and corrected if necessary. The HR per min was exported for 6s following the stimulus, so it could be expected that a minimum of five heartbeats are included in the analysis (see Prescott et al., 1992).

Avoidance behavior was assessed via the minimal distance (in m) to the agent while passing it during the BAT. Due to technical errors during data acquisition, one participant had to be excluded from data analysis of the avoidance behavior.

For the recognition data, sensitivity (*d’)* and response criteria (β) were computed according to the method recommendations outlined by Velden (1982) and Macmillan and Creelman (1991). Sensitivity (*d*’) is the ability of individuals to correctly discriminate the presented target stimuli during the experiment, from distractors which appeared at the time of recall. The decision-making response criteria (β) refer to the individual’s decision during the memory task, which can range from a liberal to a conservative response style. Hit rate was the rate of targets presented during the VR procedure that were correctly classified as targets and false alarm rate was the rate of distractors (that were added for the recognition phase) that were incorrectly classified as targets. The sensitivity index and response criteria were calculated by first transforming the hit rate and false alarm rate probabilities into standardized *z*-scores and then computing the *d’* and β statistic values (see Sawchuk et al., 2002). Under conditions in which the hit or false alarm rate were below 0.05 or above 0.95 they were transformed into 0.05 or 0.95 before conversion to *z*-scores.

The means for each agent (CS+, CS−, NS) of the fear and contingency ratings and the behavioral avoidance measured at the three rating phases (pre acquisition, post-acquisition, post extinction) were calculated. For each physiological outcome variable (fear-potentiated startle, SCR, HR), means were calculated for the CS habituation phase, while the first four reactions and the last four reactions in the acquisition and the extinction phase were computed as the means of the beginning and the end of the acquisition and extinction phase, respectively. Evoked HR was transferred with a baseline correction of the 120 s physiological baseline-measure at the beginning of the VR experiment into the HR response.

Checking for possible differences in the fear ratings before fear acquisition, an ANOVA with the within-subject factors stimulus (CS+ vs. CS−) and agent (female vs. male) and the between-subject factors social anxiety (low vs. high) and gender (women vs. men) were used for pre-acquisition phase. In order to investigate changes in fear ratings with regard to SFC ANOVAs with the within-subject factors phase (pre vs. post-acquisition for acquisition, and post-acquisition vs. post extinction for extinction), stimulus (CS+ vs. CS−) and agent (female vs. male), and the between-subject factors social anxiety (low vs. high) and gender (women vs. men) were conducted. Measuring possible generalization effects, an ANOVA was used with the within-subject factors stimulus (CS+ vs. CS− vs. NS) and agent (female vs. male) and the between-subject factors social anxiety (low vs. high) and gender (women vs. men) during post extinction phase.

An ANOVA with the within-subject factors stimulus (CS+ vs. CS−) and agent (female vs. male) and the between-subject factors social anxiety (low vs. high) and gender (women vs. men) was used for the post acquisition phase to investigate differences regarding contingency ratings. Furthermore, an ANOVA with the within-subject factors phase (post acquisition vs. post extinction), stimulus (CS+ vs. CS−) and agent (female vs. male) and the between-subject factors social anxiety (low vs. high) and gender (women vs. men) was conducted to investigate changes regarding SFC.

Checking for possible differences during CS habituation, ANOVAs with the within-subject factors stimulus (CS+ vs. CS−) and agent (female vs. male) and the between-subject factors social anxiety (low vs. high) and gender (women vs. men) were used for each physiological outcome variable (fear-potentiated startle, SCR and HR response). In order to analyze conditioning effects regarding physiological data, ANOVAs with the within-subject factors time (beginning vs. end), stimulus (CS+ vs. CS−) and agent (female vs. male) and the between-subject factors social anxiety (low vs. high) and gender (women vs. men) were conducted for the acquisition and extinction phase. Testing for possible generalization effects, ANOVAs with the within-subject factors stimulus (CS+ vs. CS− vs. NS) and agent (female vs. male) and the between-subject factors social anxiety (low vs. high) and gender (women vs. men) were conducted for the physiological variables as well.

For the behavioral avoidance data, ANOVAs with the within-subject factors phase (pre vs. post-acquisition for acquisition, and post-acquisition vs. post extinction for extinction), stimulus (CS+ vs. CS−) and agent (female vs. male) and the between-subject factors social anxiety (low vs. high) and gender (women vs. men) were conducted.

For the recognition data, ANOVAs with the within-subject factors stimulus (agent vs. object and CS+ vs. CS− vs. NS) and the between-subject factor social anxiety (low vs. high) were conducted for the *d’* and β parameter.

In follow-up analyses of significant effects of time, stimulus, agent and social anxiety as well as gender Student’s *t*-tests were performed. Partial η^2^ ($\eta_{p}^{2}$) scores and Cohen’s *d* were used as indices of effect size. The significance level was set at two-tailed α = 0.05.

## Results

### Primary Outcomes for Each Level for Acquisition and Extinction

#### Self Report: Fear Rating

**FIGURE 4:**
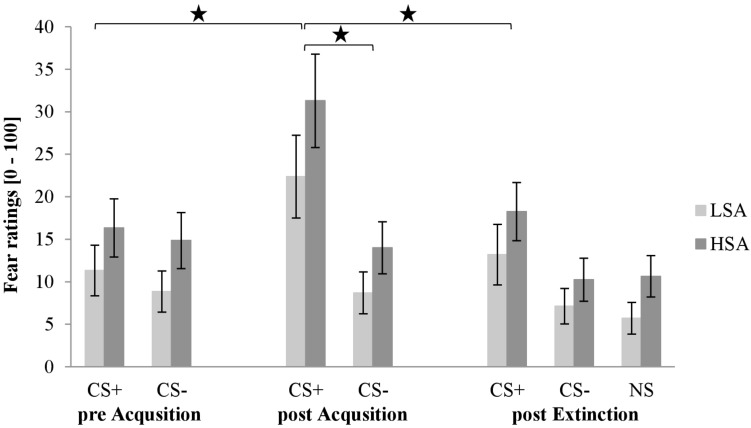
Fear ratings (*n* = 60) for CS+, CS–, and NS in the three rating phases for low (LSA) and high socially anxious (HSA) participants. CS+, agent paired with aversive unconditioned stimulus (US); CS–, agent without aversive US; NS, agent without aversive US and appearing only in the extinction phase; pre-Acquisition, after CS habituation phase; post Acquisition, after acquisition phase; post Extinction, after extinction phase. Mean fear ratings (0 = very low fear to 100 = very high fear) were given. Significant differences are indicated with an asterisk. Standard errors are presented by error bars.

Figure 4 shows the fear ratings after CS habituation, acquisition, and extinction. As we can see in the first rating, both stimuli are rated almost equal, but higher for HSA than for LSA. After the acquisition phase, fear ratings for CS+ are clearly higher than for CS−. After the extinction phase, fear ratings for CS+ decrease. However, fear ratings for CS− and NS do not differ in the third rating, whereas the CS+ is rated slightly higher than CS− and NS in both groups.

For acquisition an ANOVA comparing fear ratings before and after acquisition confirmed a significant effect of Phase × Stimulus × Gender × Social Anxiety (please see Table 1 for all significant results of the ANOVA). To disentangle this fourfold interaction further, follow-up ANOVA was conducted for LSA and HSA participants separately. For LSA, we detected significant interaction effects for Phase × Stimulus and Phase × Gender. For the Phase × Stimulus interaction, the follow-up *t*-test showed that the subjective ratings increased significantly for CS+, *t*(30) = −4.37, *p* < 0.001, *d* = 0.50, but not for CS− as expected. Follow-up *t*-test for the Phase × Gender interaction presented that fear ratings increased significantly for women, *t*(15) = −3.21, *p* = 0.006, *d* = 0.45, but not for men, which point to an enhanced fear conditioning in women compared to men. For HSA, a significant effect of Phase × Stimulus × Gender could be detected. A follow-up ANOVA was conducted for HSA women and men separately. For HSA women, a significant interaction effect of Phase × Stimulus could be detected, and the follow-up *t*-test revealed that fear ratings increased significantly for CS+, *t*(14) = −3.60, *p* = 0.003, *d* = 0.81, but not for CS−. As compared to HSA men, only a marginally significant interaction effect of Phase x Stimulus, *F*(1,56) = 3.56, *p* = 0.082,$\eta_{p}^{2}$ = 22, was found. Therefore, the fear rating results indicate that successful SFC took place for both LSA and HSA groups.

For extinction an ANOVA comparing fear ratings before and after extinction confirmed significant effects of Stimulus × Gender, Phase × Gender, Phase × Stimulus × Agent (please see Table 1 for all significant results of the ANOVA). To disentangle this threefold interaction, a follow-up ANOVA was conducted for female and male agents separately. For female agents, a significant interaction effect of Phase × Stimulus could be detected and the follow-up *t*-test revealed that the female CS+, *t*(59) = 5.93, *p* < 0.001, *d* = 0.51, and CS−, *t*(59) = 2.08, *p* = 0.042, *d* = 0.12, significantly decreased. For male agents, we identified also a significant interaction effect of Phase × Stimulus and the follow-up *t*-test highlighted that the male CS+, *t*(59) = 4.93, *p* < 0.001, *d* = 0.39, and CS−, *t*(59) = 2.88, *p* = 0.006, *d* = 0.26, significantly decreased. For the Stimulus × Gender interaction, a follow-up *t*-test showed that women rated the CS+ significantly higher than men, *t*(54.146) = 2.34, *p* = 0.023, *d* = 0.61, however there was no difference for the CS−. For the Phase × Gender interaction, a follow-up *t*-test revealed that women reported significantly more fear after the acquisition phase, *t*(53.504) = 2.33, *p* = 0.023, *d* = 0.61, than men, but not after the extinction phase. The fear rating results indicate that social fear extinction was also successful in the sample. However, no generalization effect was found.

#### Physiology: Fear-Potentiated Startle

**FIGURE 5:**
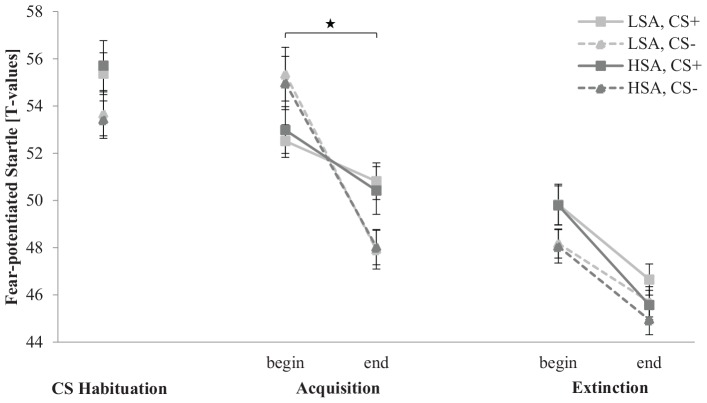
Fear-potentiated startle response (*n* = 60) for CS+ and CS– in the three phases (CS habituation, acquisition, and extinction) for low (LSA) and high social anxiety (HSA). CS+, agent paired with aversive unconditioned stimulus (US); CS–, agent without aversive US. Mean fear-potentiated startles (presented in *T*-values) were given. Significant differences are indicated with an asterisk. Standard errors are presented by error bars.

The fear-potentiated startle response for the CS habituation, acquisition, and extinction phase is depicted in Figure 5. In the CS habituation phase, the fear-potentiated startle response is slightly higher for CS+ than for CS− in both groups. In the acquisition phase, the fear-potentiated startle response for CS+ is lower than for CS− at the beginning, but higher at the end, indicating that habituation to CS− is stronger than to CS+ during SFC. In the extinction phase, the fear-potentiated startle response to both stimuli decreases from the beginning to the end in both groups.

An ANOVA investigating differences in the CS habituation phase confirmed a significant main effect of Stimulus, *F*(1,54) = 5.59, *p* = 0.022, $\eta_{p}^{2}$ = 0.09, with a higher fear-potentiated startle response for CS+ (*M* = 55.3, *SD* = 5.17) than for CS− (*M* = 53.6, *SD* = 4.87).

For the acquisition phase, an ANOVA confirmed significant effects of Time, *F*(1,52) = 42.3, *p* < 0.001, $\eta_{p}^{2}$ = 0.45, Agent × Social Anxiety, *F*(1,52) = 5.65, *p* = 0.021, $\eta_{p}^{2}$ = 0.10, Time × Stimulus, *F*(1,52) = 18.5, *p* < 0.001, $\eta_{p}^{2}$ = 0.26, and Time × Stimulus × Agent, *F*(1,52) = 9.69, *p* = 0.003, $\eta_{p}^{2}$ = 0.16. For the Agent × Social Anxiety interaction, a follow-up *t*-test revealed for LSA no significant differences between female and male agents (*p* = 0.098) and also not for HSA participants (*p* = 0.139). For the Time × Stimulus × Agent interaction, a follow-up ANOVA was conducted for female and male agents. For female agents, a significant effect of Time, *F*(1,55) = 27.0, *p* < 0.001, $\eta_{p}^{2}$ = 0.33, indicates a decrease in the fear-potentiated startle response from the beginning (*M* = 54.1, *SD* = 4.68) to the end (*M* = 49.3, *SD* = 4.80) of acquisition. Compared to male agents, a significant effect of Time, *F*(1,52) = 24.7, *p* < 0.001, $\eta_{p}^{2}$ = 0.32, and Time × Stimulus, *F*(1,52) = 29.9, *p* < 0.001, $\eta_{p}^{2}$ = 0.37, could be detected. Follow-up *t*-tests emphasized that the fear-potentiated startle response to the male CS+ was significantly lower than to the male CS− at the beginning of the acquisition, *t*(56) = −3.65, *p* < 0.001, *d* = 0.70, whereas the response to the male CS+ was significantly higher compared to the male CS− at the end of the acquisition, *t*(56) = 4.10, *p* < 0.001, *d* = 0.66. This indicates that according to the fear-potentiated startle, SFC to male agents was successfully in both groups, whereas the female CS+ and CS− did not significantly differ during fear acquisition. These results suggest enhanced fear responses and enhanced social fear conditionability to male compared to female agents.

For the extinction phase, an ANOVA found significant effects of Time, *F*(1,56) = 37.1, *p* < 0.001, $\eta_{p}^{2}$ = 0.40, Stimulus, *F*(1,56) = 6.75, *p* = 0.012, $\eta_{p}^{2}$ = 0.11, Time × Stimulus × Gender, *F*(1,56) = 4.67, *p* = 0.035, $\eta_{p}^{2}$ = 0.08, and Time × Agent × Gender, *F*(1,56) = 4.89, *p* = 0.031, $\eta_{p}^{2}$ = 0.08. To unravel this threefold interaction further, a follow-up ANOVA was conducted for each gender separately. For women, the ANOVA confirmed a significant effect of Time, *F*(1,29) = 17.6, *p* < 0.001, $\eta_{p}^{2}$ = 0.38, which indicates a further decrease in the fear-potentiated startle response from the beginning (*M* = 48.8, *SD* = 3.41) to the end (*M* = 45.5, *SD* = 3.05) of extinction. For men, the ANOVA affirmed significant effects of Time, *F*(1,27) = 20.4, *p* < 0.001, $\eta_{p}^{2}$ = 0.43, Stimulus, *F*(1, 27) = 17.6, *p* = 0.040, $\eta_{p}^{2}$ = 0.15, and Time × Stimulus, *F*(1,27) = 4.19, *p* = 0.050, $\eta_{p}^{2}$ = 0.13. The follow-up *t*-test revealed that the CS+ was significantly higher than the CS− at the beginning, *t*(28) = 2.68, *p* = 0.012, *d* = 0.76, but no more at the end of the extinction phase (*p* = 0.725). The fear-potentiated startle results indicate that social fear extinction was successful in both gender groups.

Regarding generalization effects, an ANOVA found significant effects of Stimulus, *F*(2,112) = 4.60, *p* = 0.012, $\eta_{p}^{2}$ = 0.08, Agent, *F*(1,56) = 4.81, *p* = 0.012, $\eta_{p}^{2}$ = 0.08, and Stimulus × Agent, *F*(2,112) = 5.93, *p* = 0.004, $\eta_{p}^{2}$ = 0.10. A follow-up *t*-test showed that the fear-potentiated startle response to male NS is significantly higher than to CS+, *t*(28) = −2.570, *p* = 0.013, *d* = 0.47, and CS−, *t*(59) = −5.076, *p* < 0.001, *d* = 0.84, at the beginning of the extinction phase. However, the startle response to the female CS+ is significantly higher than to the CS−, *t*(59) = 2.328, *p* = 0.023, *d* = 0.38, but not in comparison to the NS, *t*(59) = 1.465, *p* = 0.148. No clear generalization effect results were found.

#### Behavior: Behavioral Avoidance Test

**FIGURE 6:**
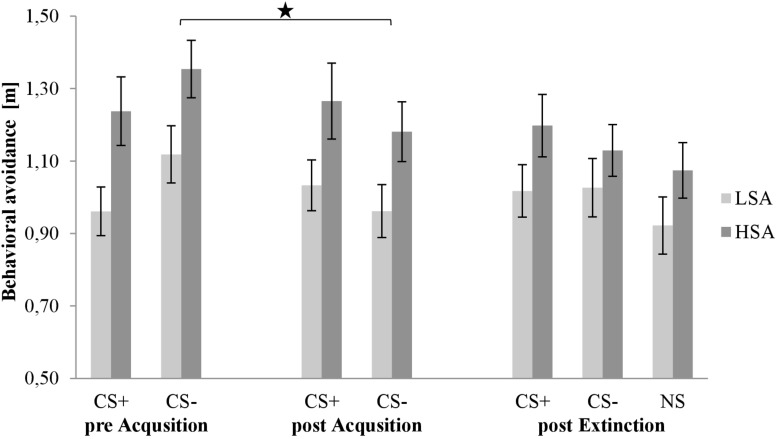
Behavioral avoidance (*n* = 59) for CS+, CS–, and NS in the three rating phases for low (LSA) and high socially anxious (HSA) participants. CS+, agent paired with aversive unconditioned stimulus (US); CS–, agent without aversive US; NS, agent without aversive US and appearing only in the extinction phase; pre-Acquisition, after CS habituation phase; post Acquisition, after acquisition phase; post Extinction, after extinction phase. Mean behavioral avoidance (presented in meter) was given. Significant differences are indicated with an asterisk. Standard errors are presented by error bars.

Figure 6 shows behavioral avoidance during all three BATs in the rating phases. As we can see in all phases, the distance to both stimuli is larger for HSA than for LSA participants. Furthermore, we can observe a small increase for the CS+ and a decrease for the CS− during SFC in both groups. After the extinction phase, the NS is nearly on the same level as the CS−.

For acquisition, an ANOVA comparing behavioral avoidance before and after acquisition confirmed significant effects of Phase × Stimulus, *F*(1,48) = 12.2, *p* < 0.001,$\eta_{p}^{2}$ = 0.20, and Stimulus × Agent × Social Anxiety, *F*(1,48) = 4.80, *p* = 0.033, $\eta_{p}^{2}$ = 0.09. A follow-up ANOVA was conducted for both social anxiety groups separately. For LSA, a significant interaction effect of Phase × Stimulus, *F*(1,25) = 8.70, *p* = 0.007, $\eta_{p}^{2}$ = 0.26, and Stimulus × Agent, *F*(1,25) = 5.26, *p* = 0.031, $\eta_{p}^{2}$ = 0.17, was found. For the Phase × Stimulus interaction, a follow-up *t*-test showed no significant change in the distance to CS+, but a significant decrease in the distance to CS−, *t*(28) = 2.756, *p* = 0.010, *d* = 0.43, from pre to post acquisition. The follow-up *t*-test for the Stimulus × Agent interaction presented no significant difference between male and female CS+ or CS−. For HSA, a significant interaction effect of Phase × Stimulus, *F*(1,23) = 4.44, *p* = 0.046, $\eta_{p}^{2}$ = 0.16, and Phase × Agent × Gender, *F*(1,23) = 4.72, *p* = 0.040, $\eta_{p}^{2}$ = 0.17, was found. A follow-up ANOVA was conducted for both genders. For HSA men, a significant interaction effect of Phase × Stimulus, *F*(1,11) = 5.67, *p* = 0.036, $\eta_{p}^{2}$ = 0.34, was found and the follow-up *t*-test revealed no significant change in the distance to CS+, but a significant decrease in the distance to CS−, *t*(12) = 2.202, *p* = 0.048, *d* = 0.49, from pre to post acquisition. For HSA women, a significant interaction effect of Phase × Agent, *F*(1,12) = 5.61, *p* = 0.035, $\eta_{p}^{2}$ = 0.32, was detected and the follow-up *t*-test showed no significant change in the distance to male CS, but a significant decrease in the distance to female CS, *t*(14) = 2.694, *p* = 0.017, *d* = 0.41, from pre to post acquisition. This indicates that HSA women maintained a larger distance to male compared to female agents, but no such differences were found for HSA men. Thus, the behavioral avoidance results indicate that successful SFC took place for both groups.

For extinction, an ANOVA comparing behavioral avoidance before and after extinction confirmed a significant effect of Phase × Stimulus × Gender, *F*(1,46) = 5.13, *p* = 0.028, $\eta_{p}^{2}$ = 0.10. A follow-up ANOVA was conducted for both genders separately. For men, a significant interaction effect of Phase × Stimulus, *F*(1,25) = 5.92, *p* = 0.022, $\eta_{p}^{2}$ = 0.19, was observed and the follow-up *t*-test revealed a marginally significant decrease in the distance to CS+, *t*(27) = 1.933, *p* = 0.064, but not to CS− from pre to post extinction. For women, no significant main or interaction effect was found. Therefore, the behavioral avoidance results show successful fear extinction for men.

**FIGURE 7:**
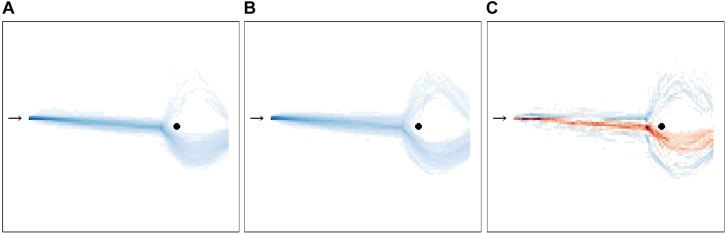
Bird’s eye view on the movement behavior (*n* = 59) during all ratings and behavioral avoidance tests (BAT). The heatmap shows the movement behavior for low **(A)** and for high socially anxious **(B)** participants. Participants started at one end of the room (black arrow) and had the task during the rating to approach the agent at the opposite end of the room (black circle). Following this, participants were instructed to pass the agent and to leave the room through the glass door located behind the agent during the BAT. The more intense the blue is, the more participants walked there. The third heatmap **(C)** shows the differences in group movement behavior comparing low (LSA) and high socially anxious (HSA) participants. Blue areas indicate that more HSA participants walked there, whereas red areas indicate that more LSA participants were there. The more intense a color is, the greater the relative difference between both groups. For interpretation of the references to color in this figure, the reader is referred to the web version of this article.

Furthermore, Figure 7 depicts the movement behavior of the LSA and HSA participants in the virtual environment and the differences in group movement behavior comparing LSA and HSA participants. Exploratory analysis of Figure 7 suggested that LSA passed more closely the agent than HSA participants.

#### Cognition: Recognition Memory Task

An ANOVA comparing the sensitivity index for the recognition stimulus agent and object confirmed a significant effect of Stimulus, *F*(1,58) = 18.3, *p* < 0.001, $\eta_{p}^{2}$ = 0.24, with a higher *d’* for agent (*M* = 1.78, *SD* = 0.68) than for object (*M* = 1.25, *SD* = 0.70), but no group differences were found. Comparing the sensitivity index for the agents with each other, an ANOVA revealed a significant effect of Stimulus, *F*(2,116) = 6.80, *p* = 0.002, $\eta_{p}^{2}$ = 0.11, with the highest value for NS (*M* = 2.33, *SD* = 1.14) compared to CS+ (*M* = 1.81, *SD* = 1.19) and CS− (*M* = 1.56, *SD* = 1.19), but no group differences were found.

Comparing the response criteria for the recognition stimulus agent and object, an ANOVA revealed a significant effect of Stimulus, *F*(1,58) = 101.9, *p* < 0.001, $\eta_{p}^{2}$ = 0.64, with a lower β for agent (*M* = −0.33, *SD* = 0.47) than for object (*M* = 0.38, *SD* = 0.41), and a marginally significant effect of Social Anxiety, *F*(1,58) = 3.71, *p* = 0.059, $\eta_{p}^{2}$ = 0.06. Tendentially, HSA (*M* = −0.06, *SD* = 0.48) exhibit a more conservative β index than LSA (*M* = 0.11, *SD* = 0.46). Comparing the response criteria for the agents with each other, an ANOVA showed a significant effect of Social Anxiety, *F*(1,58) = 5.45, *p* = 0.023, $\eta_{p}^{2}$ = 0.09, which revealed a more conservative index for HSA (*M* = −0.47, *SD* = 0.66) than for LSA (*M* = −0.19, *SD* = 0.64).

### Secondary Outcomes for Each Level for Acquisition and Extinction

#### Personality Traits and Dispositions

High socially anxious show a significantly higher score in social anxiety (SPIN), in anxiety in social interactional situations (SIAS), in fear of physiological arousal related sensations (ASI-3), in submissive behavior (SBS) as well as a significantly lower score in dominant behaviors (PRF-D) compared to the LSA group. Interestingly, HSA show significantly higher vigilance, but marginally significant lower cognitive avoidance than LSA as coping strategy. Furthermore, HSA report significantly stronger symptoms of cybersickness (SSQ) compared LSA. Regarding the mean of the experience of presence in VR (IPQ) and participants’ age, the groups do not differ significantly (see Table 2).

**TABLE 2:** Demographic variables and personality traits.

	**LSA**		**HSA**					
	**(*n* = 31)**		**(*n* = 29)**					
	***M***	***SD***	***M***	***SD***	***t***	***df***	***p***	***d***
Age	21.77	4.62	21.21	3.21	0.549	58	0.585	-
SPIN	11.13	4.06	27.93	8.36	–9.969	58	< 0.001	2.63
SIAS	16.45	9.58	29.76	13.2	–4.484	58	< 0.001	1.16
ASI-3	19.13	8.79	24.10	9.83	–2.069	58	0.043	0.55
PRF-D	9.77	3.78	5.34	4.03	4.392	58	< 0.001	1.12
SBS	23.10	7.94	28.69	7.71	–2.765	58	0.008	0.73
MCI-VIG	11.16	3.83	13.28	2.79	–2.430	58	0.018	0.64
MCI-CAV	10.55	3.44	8.97	3.02	1.888	58	0.064	-
SSQ	44.40	30.5	66.16	41.5	–2.325	58	0.024	0.61
IPQ	3.42	1.23	2.72	1.62	1.876	58	0.066	-

*Means (*M*) and standard deviations (*SD*) and also *t*- and *p*-values with degrees of freedom (*df*) and Cohen’s *d* as well as number of participants (*n*) are given for all participants for demographic variables and questionnaire data. SPIN, Social Phobia Inventory; SIAS, Social Interaction Anxiety Scale; ASI-3, Anxiety Sensitivity Index; PRF-D, German Personality Research Form – Dominance Scale; SBS, Submissive Behavior Scale; MCI-VIG/MCI-COV, Vigilance/Cognitive Avoidance coping strategy of the Mainz Coping Inventory; SSQ, Simulator Sickness Questionnaire; IPQ, Igroup Presence Questionnaire.*

Furthermore, there is a marginally significant negative correlation between social fear learning (difference of CS+ and CS− after acquisition) and dominance (*r* = −0.22, *p* = 0.086) as well as a significant positive correlation with submissive behavior (*r* = 0.30, *p* = 0.021).

An exploratory measure of the post questionnaire for the evaluation of the US showed that the spitting and rejection of the male, *F*(1,59) = 13.2, *p* < 0.001, $\eta_{p}^{2}$ = 19, and female agent, *F*(1,59) = 19.4, *p* < 0.001, $\eta_{p}^{2}$ = 25, caused more fear by women compared to men, but not by HSA compared to LSA.

#### Self Report: Contingency Rating

**FIGURE 8:**
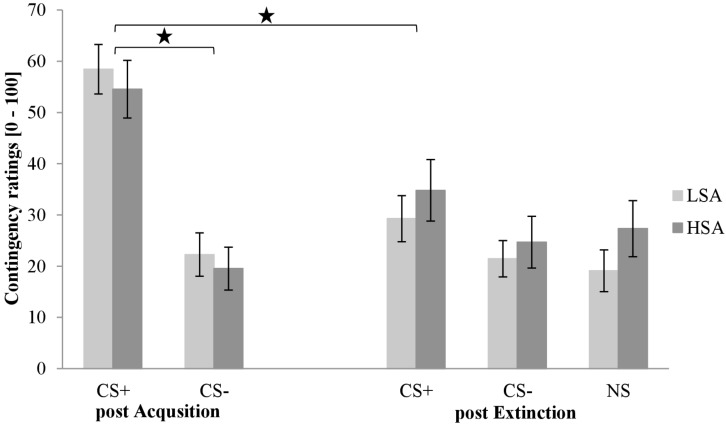
Contingency ratings (*n* = 60) for CS+, CS–, and NS in the rating phases 2 and 3 for low (LSA) and high socially anxious (HSA) participants. CS+, agent paired with aversive unconditioned stimulus (US); CS–, agent without aversive US; NS, agent without aversive US and appearing only in the extinction phase; post Acquisition, after acquisition phase; post Extinction, after extinction phase. Mean contingency ratings (0 = very unlikely to 100 = very likely) were given. Significant differences are indicated with an asterisk. Standard errors are presented by error bars.

The contingency ratings post acquisition and extinction is shown in Figure 8. As can be seen in the first rating, the CS+ is rated higher than the CS− in both groups after acquisition. In the third rating, CS+ is slightly higher than CS− and NS in both groups.

An ANOVA comparing contingency ratings after the acquisition confirmed a significant effect of Stimulus, *F*(1,56) = 74.1, *p* < 0.001, $\eta_{p}^{2}$ = 57, Gender, *F*(1,56) = 12.8, *p* < 0.001, $\eta_{p}^{2}$ = 0.19, and a marginally significant interaction effect of Stimulus × Gender, *F*(1,56) = 3.87, *p* = 0.054, $\eta_{p}^{2}$ = 0.07. An exploratory *t*-test revealed that women rated the CS+ significantly higher than men, *t*(58) = 3.83, *p* < 0.001, *d* = 1.01, whereas the CS− did not differ between genders (*p* = 0.120).

An ANOVA comparing contingency ratings before and after extinction confirmed significant effects of Phase, *F*(1,56) = 15.2, *p* < 0.001, $\eta_{p}^{2}$ = 21, Stimulus, *F*(1,56) = 82.3, *p* < 0.001, $\eta_{p}^{2}$ = 60, Agent, *F*(1,56) = 5.31, *p* = 0.025, $\eta_{p}^{2}$ = 09, Gender, *F*(1,56) = 4.50, *p* = 0.038, $\eta_{p}^{2}$ = 07, and Phase × Stimulus, *F*(1,56) = 42.5, *p* < 0.001, $\eta_{p}^{2}$ = 43, and Phase × Social Anxiety × Gender, *F*(1,56) = 5.89, *p* = 0.019, $\eta_{p}^{2}$ = 10. To unravel this threefold interaction, a follow-up ANOVA was conducted for LSA and HSA participants separately. For LSA, significant main effects for Phase, *F*(1,29) = 21.6, *p* < 0.001, $\eta_{p}^{2}$ = 38, Stimulus, *F*(1,29) = 46.6, *p* < 0.001, $\eta_{p}^{2}$ = 62, Agent, *F*(1,29) = 6.20, *p* = 0.019, $\eta_{p}^{2}$ = 18, and interaction effects of Phase × Stimulus, *F*(1,29) = 8.89, *p* = 0.006, $\eta_{p}^{2}$ = 24, and Phase × Gender, *F*(1,29) = 25.9, *p* < 0.001, $\eta_{p}^{2}$ = 47, could be detected. For the Phase × Stimulus interaction, a follow-up *t*-test showed that the subjective ratings decreased significantly for CS+, *t*(30) = 5.50, *p* < 0.001, *d* = 1.14, but not for CS−. A follow-up *t*-test for the Phase × Gender interaction showed that contingency ratings decreased significantly for women, *t*(15) = 4.92, *p* < 0.001, *d* = 1.39, but not for men. For HSA, a significant effect of Stimulus, *F*(1,27) = 36.5, *p* < 0.001, $\eta_{p}^{2}$ = 58, Gender, *F*(1,27) = 8.95, *p* = 0.006, $\eta_{p}^{2}$ = 39, and Phase × Stimulus could be detected. A follow-up *t*-test revealed that fear ratings decreased significantly for CS+, *t*(28) = 3.35, *p* = 0.002, *d* = 0.65, but not for CS−. Thus, contingency rating results also indicate that SFC was successful regarding participants’ cognitive appraisal.

#### Physiology: Skin Conductance Response

**FIGURE 9:**
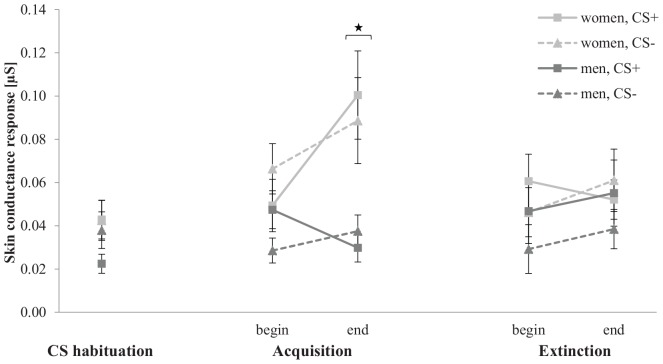
Skin conductance response (*n* = 49) for CS+ and CS– in the three phases (CS habituation, acquisition, and extinction) for both genders. CS+, agent paired with aversive unconditioned stimulus (US); CS–, agent without aversive US. Mean skin conductance response (presented in μS) was given. Significant differences are indicated with an asterisk. Standard errors are presented by error bars.

Figure 9 shows the SCR for CS habituation, acquisition, and extinction phase. In the CS habituation phase, the SCR for men is slightly lower for CS+ than for CS−, whereas women do not differ between both stimuli. In the acquisition phase, the SCR increase for women, but not for men. Furthermore, women reveal a higher increase for CS+ than for CS−. In the extinction phase, the SCR for both genders do not differ from the beginning to the end.

Proving differences in the CS habituation phase, an ANOVA confirmed no significant effects. For the acquisition, an ANOVA showed a significant effect of Time, *F*(1,45) = 5.45, *p* = 0.024, $\eta_{p}^{2}$ = 0.11, Gender, *F*(1,45) = 9.86, *p* = 0.003, $\eta_{p}^{2}$ = 0.18, Time × Gender, *F*(1,45) = 8.51, *p* = 0.005, $\eta_{p}^{2}$ = 0.16, Agent × Gender, *F*(1,45) = 5.36, *p* = 0.025, $\eta_{p}^{2}$ = 0.11, Time × Agent, *F*(1,45) = 6.33, *p* = 0.016, $\eta_{p}^{2}$ = 0.12, Time × Stimulus × Gender, *F*(1,45) = 4.71, *p* = 0.035, $\eta_{p}^{2}$ = 0.10, Time × Agent × Gender, *F*(1,45) = 6.37, *p* = 0.015, $\eta_{p}^{2}$ = 0.12. A follow-up ANOVA was conducted for both genders separately. For women, a significant effect of Time, *F*(1,20) = 7.25, *p* = 0.014, $\eta_{p}^{2}$ = 27, and Time × Agent, *F*(1,20) = 12.8, *p* = 0.002, $\eta_{p}^{2}$ = 39, could be detected. Follow-up *t*-test revealed that the SCR to the female agents significantly increase, *t*(21) = −4.14, *p* < 0.001, *d* = 0.80, whereas the SCR to the male agents did not significantly increase from the beginning to the end of the acquisition. For men, significant effects of Agent, *F*(1,25) = 6.07, *p* = 0.021, $\eta_{p}^{2}$ = 0.20, Social Anxiety, *F*(1,25) = 5.26, *p* = 0.031, $\eta_{p}^{2}$ = 0.17, Agent × Social Anxiety, *F*(1,25) = 7.10, *p* = 0.013, $\eta_{p}^{2}$ = 0.22, Time × Stimulus, *F*(1,25) = 6.18, *p* = 0.020, $\eta_{p}^{2}$ = 0.20, and Time × Stimulus × Social Anxiety, *F*(1,25) = 4.63, *p* = 0.041, $\eta_{p}^{2}$ = 0.16, were located.

According this threefold interaction, a follow-up ANOVA was conducted for male LSA and HSA participants. For LSA men, a significant effect of Agent, *F*(1,13) = 8.04, *p* = 0.014, $\eta_{p}^{2}$ = 0.38, and Time × Stimulus, *F*(1,13) = 7.14, *p* = 0.019, $\eta_{p}^{2}$ = 0.35, were found and the follow-up *t*-tests revealed that the SCR to the CS+ was significantly higher than to the CS−, *t*(13) = 2.24, *p* = 0.043, *d* = 0.70, at the beginning but no more at the end of the acquisition phase. For HSA participants, no significant main or interaction effect was found. The SCR results indicate for women higher fear responses to female compared to male agents, whereas only LSA men showed a differentiation between the CS+ and CS− at the beginning of the acquisition.

For the extinction, an ANOVA confirmed significant effects of Stimulus × Social Anxiety, *F*(1,45) = 4.04, *p* = 0.050, $\eta_{p}^{2}$ = 0.08, Agent × Social Anxiety, *F*(1,45) = 11.8, *p* < 0.001, $\eta_{p}^{2}$ = 0.21, Time × Agent, *F*(1,45) = 6.06, *p* = 0.018, $\eta_{p}^{2}$ = 0.12, and Time × Stimulus × Social Anxiety, *F*(1,45) = 4.95, *p* = 0.031, $\eta_{p}^{2}$ = 0.10. Follow-up ANOVA was conducted for both LSA and HSA participants. For LSA, a significant effect of Stimulus, *F*(1,22) = 6.26, *p* = 0.020,$\eta_{p}^{2}$ = 0.22, Agent, *F*(1,22) = 11.8, *p* = 0.002, $\eta_{p}^{2}$ = 0.35, and Time × Stimulus, *F*(1,22) = 4.90, *p* = 0.037, $\eta_{p}^{2}$ = 0.18, could be detected. Follow-up *t*-test showed that the CS+ is significantly higher compared to the CS− at the beginning of the extinction, *t*(23) = 3.63, *p* < 0.001, *d* = 0.58, but not more at the end of the extinction. For HSA participants, only a significant main effect of Agent, *F*(1,23) = 5.29, *p* = 0.031,$\eta_{p}^{2}$ = 0.19, which indicates a higher response to female (*M* = 0.062, *SD* = 0.065) compared to male agents (*M* = 0.037, *SD* = 0.03). The SCR results indicate successful extinction for LSA, whereas HSA show no consistent extinction.

#### Physiology: Heart Rate Response

**FIGURE 10:**
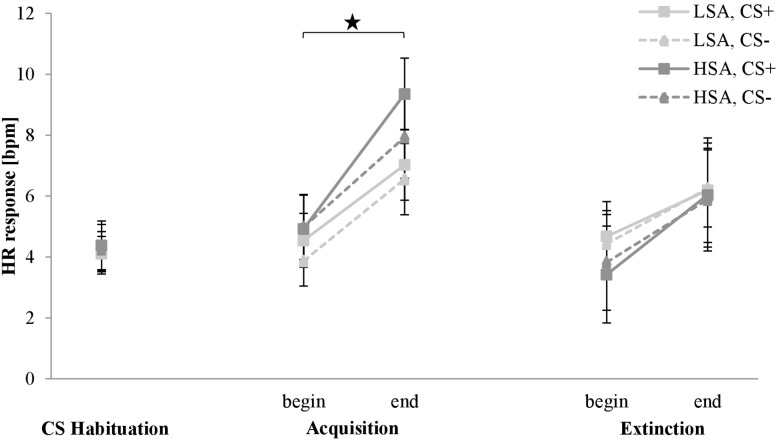
Heart rate (HR) response (*n* = 60) for CS+ and CS– in the three phases (CS habituation, acquisition, and extinction) for low (LSA) and high social anxiety (HSA). CS+, agent paired with aversive unconditioned stimulus (US); CS–, agent without aversive US. Mean HR response (presented in beats per min) was given. Significant differences are indicated with an asterisk. Standard errors are presented by error bars.

The HR response for the CS habituation, acquisition, and extinction phase is shown in Figure 10. The HR response is approximately equivalent for both stimuli in both groups in the CS habituation phase. From the beginning to the end of the acquisition phase, HR response increases for both stimuli in both groups. For HSA, HR response for CS+ is higher than for CS− at the end of the acquisition phase. In the extinction phase, HR response increases slightly for both stimuli to a comparable level from the beginning to the end in both groups.

Investigating differences in the CS habituation phase, an ANOVA confirmed no significant effects. For the acquisition phase, an ANOVA confirmed a significant effect of Time, *F*(1,52) = 42.3, *p* < 0.001, $\eta_{p}^{2}$ = 0.45, which indicates an increase from the beginning (*M* = 4.59, *SD* = 5.22) to the end (*M* = 7.75, *SD* = 6.24). The HR response results indicate only activation during the fear acquisition in both groups.

For the extinction phase, an ANOVA revealed significant effects of Time, *F*(1,55) = 41.7, *p* < 0.001, $\eta_{p}^{2}$ = 0.43, and Time × Social Anxiety × Gender, *F*(1,55) = 4.78, *p* = 0.033, $\eta_{p}^{2}$ = 0.08. A follow-up ANOVA was conducted for each group. For LSA, there was a significant effect of Time, *F*(1,20) = 6.05, *p* = 0.023, $\eta_{p}^{2}$ = 0.23, and Time × Agent × Gender, *F*(1,20) = 6.05, *p* = 0.023, $\eta_{p}^{2}$ = 0.23. A follow-up ANOVA for the significant interaction showed for men a marginally significant effect of Time × Agent, *F*(1,14) = 4.10, *p* = 0.062, $\eta_{p}^{2}$ = 0.23, and for women a significant increase from the beginning to the end of the extinction, *F*(1,15) = 11.9, *p* = 0.003, $\eta_{p}^{2}$ = 0.44. For HSA, a significant effect of Time, *F*(1,20) = 6.05, *p* = 0.023, $\eta_{p}^{2}$ = 0.23, and Time × Gender, *F*(1,20) = 6.05, *p* = 0.023, $\eta_{p}^{2}$ = 0.23, was found. A follow-up *t*-test revealed that during extinction the HR response for women increased significantly, *t*(14) = −5.621, *p* < 0.001, *d* = 0.5, but not the HR response for men (*p* = 0.292). The HR response results show higher activation during extinction for women compared to men in both groups.

## Discussion

The present study examined gender differences in low and high socially anxious participants regarding the acquisition and extinction of conditioned social fear to virtual female and male agents. Participants actively approached virtual agents using a joystick in a setting similar to the one used by Reichenberger et al. (2017). During the experiment the following primary outcome variables, which are defined for each of four levels of emotional reactions, include experience (fear ratings), psychophysiology (fear-potentiated startle), behavior (avoidance) and cognition (unforeseen recognition memory task). Secondary outcome variables were personality traits, contingency ratings, SCR and HR response.

In line with previous experiments (Shiban et al., 2015; Reichenberger et al., 2017), the applied SFC paradigm proved to effectively induce conditioned responses. After acquisition, both groups exhibited enhanced social fear responses to the CS+ compared to the CS− agent. Such successful social fear conditioning was reflected by our primary outcome variables, e.g., fear ratings, fear-potentiated startle, and behavioral avoidance.

Consistent with our hypotheses, fear ratings clearly increased for CS+ compared to CS− after the acquisition phase and fear conditioning was enhanced in women compared to men in both groups. Interestingly, there were no increased fear ratings to CS− agents after acquisition in HSA compared to LSA participants. Furthermore, we found no enhanced fear ratings when approaching male agents in comparison to female agents.

Regarding the primary variable within the physiological measures, a clear difference in the fear-potentiated startle between male CS+ and CS− at the end of the acquisition indicates a successful SFC to male agents in both groups. However, the fear-potentiated startle response to both female CS+ and CS− agents habituated during acquisition. Thus, primary physiological results indicate enhanced fear responses and enhanced social fear conditionability to male than to female agents.

Concerning the primary behavioral outcome, we found a generally increased avoidance to agents in HSA compared to LSA participants in the BATs. Furthermore, there was no significant change in the distance to CS+ before and after acquisition, but a decrease in the distance to CS− in both groups. In line with Iachini et al. (2014), we found a larger distance to male compared to female agents in fear conditioning for HSA women. Interestingly, HSA men do not seem to differentiate between a female or male aversive person.

The subsequently conducted recognition memory task revealed that participants replied with higher sensitivity for agent than for object stimuli. Therefore, we could conclude that the ability of the participants to correctly distinguish between the target and distractor was better for social (agents) compared to neutral (objects) cues and that the attention was focused more on social cues. Furthermore, we found the highest sensitivity for NS compared to CS+ and CS− stimuli. Concerning the social cue, participants were better at distinguishing the target from the distractor for the NS compared to CS+ and CS− agent. Regarding the decision-making response criterion, HSA exhibited a more conservative response style than LSA participants, which means that HSA tend to avoid a false alarm and give rather the response No than Yes. In general, participants showed a more conservative response style for agent than for object stimuli. Furthermore, HSA responded in a more conservative manner than LSA participants by comparing the agents with each other.

Concerning the secondary outcome variables, women estimated the appearance of the US to be more likely in the presence of a CS+ agent than men reflected in the contingency ratings. With regard to HR response, there was activation from the beginning to the end of acquisition, but no differentiation between the stimuli or groups. Moreover, we observed higher SCR in women at the end of acquisition compared to men, but no CS+/CS− differences were revealed. Our SCR and HR response results were only partly in concordance with the primary outcome variables. First of all, an attenuated physiological change following conditioning due to high interindividual differences in fear responses on the physiological level is reported frequently (Craske et al., 1991). Secondly, the segmented time period for SCR and HR response were 6 s after onset approaching the agent, but due to the startle noise at about 6, 5–7 s, it was not possible to analyze the interesting uncertain “second half” approach toward the agent. Following this, we assume that in the first half of the approach participants exhibited a lower sensitivity of fear responses.

Regarding the personality traits and dispositions, we found that rather submissive participants showed enhanced social fear learning, whereas rather dominant participants exhibited a tendency for reduced social fear learning. Regarding the coping strategies, HSA participants reported higher vigilance and lower cognitive avoidance as strategies they usually use in comparison to LSA. Vigilance and cognitive avoidance refer to changes in attentional orientation and information processing in situations where individuals are confronted with threat-related information (Krohne et al., 2000). Following our results, HSA persons are more likely to avert attention from threat-relevant stimuli (e.g., aversive agents, CS+) and to inhibit further processing of such information. In contrast, HSA show an increased intake and comprehensive processing of threatening information. Krohne et al. (2000) suggest these two tendencies as independently varying personality dimensions, and assume that emotional arousal triggers the tendency to cognitive avoidance of threat-related cues (control of anxiety), while uncertainty activates vigilant behavioral tendencies (control of danger). Measuring attentional orientation and information processing in social threat-related situations on a behavioral level, it would be interesting to analyze the gaze of low and high socially anxious via an eye-tracking method.

Regarding fear extinction in the fear and contingency ratings, the variation between the CS+ and CS− that followed acquisition vanished after the extinction phase for both groups. Interestingly, comparing fear ratings before and after extinction in HSA, ratings for CS+ were higher by women than by men, suggesting higher resistance to extinction in women compared to men. However, we found no stronger fear of CS+ agents during fear extinction in HSA compared to LSA. The fear-potentiated startle reflected a further habituation for CS+ and CS− stimuli, indicating successful social fear extinction. No generalization effect was reflected by the subjective and physiological measures.

In the present study, a SPIN cut-off value of 19 was used to distinguish between LSA and HSA participants. According to Sosic et al. (2008) a cut-off score of 25 provides the optimal basis for differentiating between individuals with SAD and healthy controls. Thus, our HSA sample could not be regarded as a clear clinical sample. However, the cut-off value of 19 is sufficient to secure a sample of participants that have significant higher social fear than average persons (Connor et al., 2000). The sample, which consisted mainly of young students, might be another limitation that should be considered when generalizing the results to a broader context.

Besides the higher social fear conditionability in women, the menstrual cycle phase might be another factor influencing conditioning and extinction of social fear. Using a 2-day fear conditioning and extinction protocol, Milad et al. (2006) reported reduced extinction memory among women in the late follicular cycle phase in comparison to women in the early follicular phase and men. Thus, hormones prevailing in the late follicular cycle phase might attenuate extinction recall. The effect of particular hormones in different cycle phases on social fear learning and extinction could also be investigated in a larger female sample using the SFC paradigm in VR in the future.

Furthermore, individual factors, such as personality traits and dispositions, should be emphasized more in future SFC research to better understand their predictive influence for developing SAD (Beckers et al., 2013). For example, we included individual submissive and dominance behavior as well as coping mechanisms, e.g., vigilance and cognitive avoidance, in a first step for a broader investigation. In future, we will measure coping strategies and individual dispositions, e.g., submissive and dominance, with more regard to the presented social interaction for a more comprehensive examination.

## Conclusion

Our SFC paradigm is a useful tool to investigate the learning and unlearning of social fear in a VR setting with enhanced ecological validity. Regarding fear ratings, women showed an enhanced fear conditioning compared to men, but we found no enhanced fear ratings when approaching male compared to female agents. Furthermore, concerning the contingency ratings women also estimated the appearance of the US to be more likely in the presence of a CS+ agent compared to men. Interestingly, the fear-potentiated startle reflected enhanced fear responses and enhanced social fear conditionability in male compared to female agents. Additionally, HSA women showed a larger behavioral avoidance to male compared to female agents in fear conditioning, whereas HSA men do not seem to differentiate between female or male aversive persons. Interestingly, participants’ gender was relevant for measures prone to be influenced by reflective processes (fear and contingency ratings as well as SCR), while more automatic measures like fear-potentiated startle and also behavioral avoidance seem to depend on the gender of the virtual agent. Our SFC paradigm aims to achieve a deeper understanding of the underlying mechanisms of development and maintenance of social anxiety in men and women. Altogether, the present research is one of few examples of a fear conditioning paradigm with social interactions in a standardized and experimentally controlled way, with a disorder-relevant US to examine affective learning, and with a high ecological validity. VR allows more interactive experimental designs which increase the realism of the social interaction and can reveal enhanced behavioral responses (Wieser et al., 2009). Thus, empirical research on social anxiety can benefit from using VR. Our study is the first to investigate the mentioned conditioning effect in a paradigm that aims – in contrast to classical paradigms – to enhance ecological validity while securing experimental control. Furthermore, we included approach behavior to anthropomorphic agents as a feature as well as a dependent variable. Moreover, we involved independent variables such as the gender of participants, gender of agents, and social anxiety in one study and thus could investigate interaction effects between these factors. Furthermore, we consider the novelty and the strength of our study in introducing a VR paradigm, which is a promising tool to measure emotional learning processes on each of the four levels of emotional reactions including experience, psychophysiology, behavior, and cognition with an enhanced ecological validity. Further research is needed to expand our findings by increasing a more heterogeneous sample and by testing individuals suffering from SAD. In addition, next steps could be the involvement of the oxytocin system (Zoicas et al., 2014; Neumann and Slattery, 2016) and including eye-tracking analysis (Mühlberger et al., 2008; Wieser et al., 2009; Dechant et al., 2017).

## Ethics Statement

This study was carried out in accordance with the recommendations of the Ethics Committee of the University of Regensburg with written informed consent from all subjects. All subjects gave written informed consent in accordance with the Declaration of Helsinki. The protocol was approved by the Ethics Committee of the University of Regensburg.

## Author Contributions

JR conceived the study, data acquisition and analysis, and wrote the manuscript. MP analyzed the data and contributed to writing the manuscript. DF acquired and analyzed the data and contributed to writing the manuscript. JG acquired and analyzed the data, and contributed to writing the manuscript. YS contributed to writing the manuscript. AM contributed to study conception, analyzed the data, and contributed to writing the manuscript. All authors approved the final version of the manuscript for submission.

## Conflict of Interest Statement

AM is stakeholder of a commercial company that develops and sells virtual environment research systems. The remaining authors declare that the research was conducted in the absence of any commercial or financial relationships that could be construed as a potential conflict of interest. The reviewer RM declared a shared affiliation, with no collaboration, with the authors to the handling Editor at the time of review.

## Footnotes

1 https://github.com/ValveSoftware/openvr

## References

[B1] Ahrens L. M. Mühlberger A. Pauli P. Wieser M. J. (2015). Impaired visuocortical discrimination learning of socially conditioned stimuli in social anxiety. *Soc. Cogn. Affect. Neurosci.* 10 929–937. 10.1093/scan/nsu140 25338634 PMC4483562

[B2] Ahrens L. M. Pauli P. Reif A. Mühlberger A. Langs G. Aalderink T. (2016). Fear conditioning and stimulus generalization in patients with social anxiety disorder. *J. Anxiety Disord.* 44 36–46. 10.1016/j.janxdis.2016.10.003 27728838

[B3] Åhs F. Dunsmoor J. E. Zielinski D. LaBar K. S. (2015). Spatial proximity amplifies valence in emotional memory and defensive approach-avoidance. *Neuropsychologia* 70 476–485. 10.1016/j.neuropsychologia.2014.12.018 25541499 PMC4402246

[B4] Allan S. Gilbert P. (1997). Submissive behaviour and psychopathology. *Br. J. Clin. Psychol.* 36 467–488. 10.1111/j.2044-8260.1997.tb01255.x 9403141

[B5] American Psychiatric Association [APA] (2013). *Diagnostic and Statistical Manual of Mental Disorders*, 5th Edn Washington, D.C.: American Psychiatric Publishing.

[B6] Andreatta M. Neueder D. Glotzbach-Schoon E. Mühlberger A. Pauli P. (2017). Effects of context preexposure and delay until anxiety retrieval on generalization of contextual anxiety. *Learn. Mem.* 24 43–54. 10.1101/lm.044073.116 27980075 PMC5159654

[B7] Beckers T. Krypotos A.-M. Boddez Y. Effting M. Kindt M. (2013). What’s wrong with fear conditioning? *Biol. Psychol.* 92 90–96. 10.1016/j.biopsycho.2011.12.015 22223096

[B8] Blumenthal T. D. Cuthbert B. N. Filion D. L. Hackley S. Lipp O. V. Van Boxtel A. (2005). Committee report: guidelines for human startle eyeblink electromyographic studies. *Psychophysiology* 42 1–15. 10.1111/j.1469-8986.2005.00271.x 15720576

[B9] Cartaud A. Ruggiero G. Ott L. Iachini T. Coello Y. (2018). Physiological response to facial expressions in peripersonal space determines interpersonal distance in a social interaction context. *Front. Psychol.* 9:657. 10.3389/fpsyg.2018.00657 29867639 PMC5949865

[B10] Clark D. M. Wells A. (1995). “A cognitive model of social phobia,” in *Social Phobia: Diagnosis, Assessment, and Treatment*, eds Heimberg R. G. Liebowitz M. Hope D. A. Schneier F. (New York, NY: Guilford), 69–93.

[B11] Connor K. M. Davidson J. R. Churchill L. E. Sherwood A. Weisler R. H. Foa E. (2000). Psychometric properties of the social phobia inventory (SPIN): new self-rating scale. *Br. J. Psychiatry* 176 379–386. 10.1192/bjp.176.4.379 10827888

[B12] Craske M. G. Street L. L. Jayaraman J. Barlow D. H. (1991). Attention versus distraction during in vivo exposure: snake and spider phobias. *J. Anxiety Disord.* 5 199–211. 10.1016/0887-6185(91)90001-A

[B13] Dechant M. Trimpl S. Wolff C. Mühlberger A. Shiban Y. (2017). Potential of virtual reality as a diagnostic tool for social anxiety: a pilot study. *Comput. Hum. Behav.* 76 128–134. 10.1016/j.chb.2017.07.005

[B14] Diemer J. Alpers G. W. Peperkorn H. M. Shiban Y. Mühlberger A. (2015). The impact of perception and presence on emotional reactions: a review of research in virtual reality. *Front. Psychol.* 6:26. 10.3389/fpsyg.2015.00026 25688218 PMC4311610

[B15] Duits P. Cath D. C. Lissek S. Hox J. J. Hamm A. O. Engelhard I. M. (2015). Updated meta-analysis of classical fear conditioning in the anxiety disorders. *Depress. Anxiety* 32 239–253. 10.1002/da.22353 25703487

[B16] Dymond S. Schlund M. W. Roche B. De Houwer J. Freegard G. P. (2012). Safe from harm: learned, instructed, and symbolic generalization pathways of human threat-avoidance. *PLoS One* 7:e47539. 10.1371/journal.pone.0047539 23077631 PMC3471858

[B17] Fehm L. Pelissolo A. Furmark T. Wittchen H.-U. (2005). Size and burden of social phobia in Europe. *Eur. Neuropsychopharmacol.* 15 453–462. 10.1016/j.euroneuro.2005.04.002 15921898

[B18] Foa E. B. Kozak M. J. (1986). Emotional processing of fear: exposure to corrective information. *Psychol. Bull.* 99 20–35. 10.1037/0033-2909.99.1.20 2871574

[B19] Gilboa-Schechtman E. Freshman M. Amir N. Foa E. (1997). “Have I seen this face before? memory for facial expressions in generalized social phobics,” in *Proceedings of the Thirty-first Annual Conference of the Association for the Advancement of Behavior Therapy*, New York, NY.

[B20] Glotzbach E. Ewald H. Andreatta M. Pauli P. Mühlberger A. (2012). Contextual fear conditioning predicts subsequent avoidance behaviour in a virtual reality environment. *Cogn. Emot.* 26 1256–1272. 10.1080/02699931.2012.656581 22551520

[B21] Glotzbach-Schoon E. Andreatta M. Mühlberger A. Pauli P. (2013). Context conditioning in virtual reality as a model for pathological anxiety. *Neuroforum* 19 110–117. 10.1007/s13295-013-0047-z

[B22] Hamm A. O. Vaitl D. Lang P. J. (1989). Fear conditioning, meaning, and belongingness: a selective association analysis. *J. Abnorm. Psychol.* 98 395–406. 10.1037/0021-843X.98.4.395 2592673

[B23] Heimberg R. G. Turk C. L. (2002). “Assessment of social phobia,” in *Cognitive-Behavioral Group Therapy for Social Phobia: Basic Mechanisms and Clinical Strategies*, eds Heimberg R. G. Becker R. E. (New York, NY: Guilford), 107–126.

[B24] Heinrichs N. Hahlweg K. Fiegenbaum W. Frank M. Schroeder B. Von Witzleben I. (2002). Validität und reliabilität der social interaction anxiety scale (SIAS) und der social phobia scale (SPS). *Verhaltenstherapie* 12 26–35. 10.1159/000056690

[B25] Hermann C. Ziegler S. Birbaumer N. Flor H. (2002). Psychophysiological and subjective indicators of aversive pavlovian conditioning in generalized social phobia. *Biol. Psychiatry* 52 328–337. 10.1016/s0006-3223(02)01385-9 12208640

[B26] Hofmann S. G. (2007). Cognitive factors that maintain social anxiety disorder: a comprehensive model and its treatment implications. *Cogn. Behav. Ther.* 36 193–209. 10.1080/16506070701421313 18049945 PMC2151931

[B27] Hofmann S. G. Alpers G. Pauli P. (2009). “Phenomenology of panic and phobic disorders,” in *Oxford Handbook of Anxiety and Related Disorders*, eds Antony M. M. Stein M. B. (New York, NY: Oxford University Press), 34–46.

[B28] Huff N. C. Zielinski D. J. Fecteau M. E. Brady R. LaBar K. S. (2010). Human fear conditioning conducted in full immersion 3-dimensional virtual reality. *J. Vis. Exp.* 42:1993. 10.3791/1993 20736913 PMC3278330

[B29] Iachini T. Coello Y. Frassinetti F. Ruggiero G. (2014). Body space in social interactions: a comparison of reaching and comfort distance in immersive virtual reality. *PLoS One* 9:e111511. 10.1371/journal.pone.0111511 25405344 PMC4236010

[B30] Iachini T. Coello Y. Frassinetti F. Senese V. P. Galante F. Ruggiero G. (2016). Peripersonal and interpersonal space in virtual and real environments: effects of gender and age. *J. Environ. Psychol.* 45 154–164. 10.1016/j.jenvp.2016.01.004

[B31] Jackson E. D. Payne J. D. Nadel L. Jacobs W. J. (2006). Stress differentially modulates fear conditioning in healthy men and women. *Biol. Psychiatry* 59 516–522. 10.1016/j.biopsych.2005.08.002 16213468

[B32] Kemper C. J. Ziegler M. Taylor S. (2009). Überprüfung der psychometrischen qualität der deutschen version des angstsensitivitätsindex-3. *Diagnostica* 55 223–233. 10.1026/0012-1924.55.4.223

[B33] Kennedy R. S. Lane N. E. Berbaum K. S. Lilienthal M. G. (1993). Simulator sickness questionnaire: an enhanced method for quantifying simulator sickness. *Int. J. Aviat. Psychol.* 3 203–220. 10.1207/s15327108ijap0303_3

[B34] Kinateder M. Gromer D. Gast P. Buld S. Müller M. Jost M. (2015). The effect of dangerous goods transporters on hazard perception and evacuation behavior – A virtual reality experiment on tunnel emergencies. *Fire Saf. J.* 78 24–30. 10.1016/j.firesaf.2015.07.002

[B35] Krohne H. W. Egloff B. (1999). *Das Angstbewältigungs-Inventar (ABI).* Frankfurt: Swets Test Services GmbH.

[B36] Krohne H. W. Egloff B. Varner L. J. Burns L. R. Weidner G. Ellis H. C. (2000). The assessment of dispositional vigilance and cognitive avoidance: factorial structure, psychometric properties, and validity of the mainz coping inventory. *Cogn. Ther. Res.* 24 297–311. 10.1023/A:1005511320194

[B37] Lang P. J. (1968). “Fear reduction and fear behavior: problems in treating a construct,” in *Research in Psychotherapy*, ed. Shlien J. M. (Washington, DC: American Psychological Association), 90–103.

[B38] Lipp O. V. Siddle D. A. Dall P. J. (2003). The effects of unconditional stimulus valence and conditioning paradigm on verbal, skeleto-motor, and autonomic indices of human Pavlovian conditioning. *Learn. Motiv.* 34 32–51. 10.1016/s0023-9690(02)00507-6

[B39] Lissek S. Grillon C. (2010). Overgeneralization of conditioned fear in the anxiety disorders. *J. Psychol.* 218 146–148. 10.1027/0044-3409/a000022

[B40] Lissek S. Levenson J. Biggs A. L. Johnson L. L. Ameli R. Pine D. S. (2008). Elevated fear conditioning to socially relevant unconditioned stimuli in social anxiety disorder. *Am. J. Psychiatry* 165 124–132. 10.1176/appi.ajp.2007.06091513 18006874 PMC2538574

[B41] Lissek S. Pine D. S. Grillon C. (2006). The strong situation: a potential impediment to studying the psychobiology and pharmacology of anxiety disorders. *Biol. Psychol.* 72 265–270. 10.1016/j.biopsycho.2005.11.004 16343731

[B42] Lissek S. Powers A. S. McClure E. B. Phelps E. A. Woldehawariat G. Grillon C. (2005). Classical fear conditioning in the anxiety disorders: a meta-analysis. *Behav. Res. Ther.* 43 1391–1424. 10.1016/j.brat.2004.10.00 15885654

[B43] Lonsdorf T. B. Menz M. M. Andreatta M. Fullana M. A. Golkar A. Haaker J. (2017). Don’t fear ‘fear conditioning’: Methodological considerations for the design and analysis of studies on human fear acquisition, extinction, and return of fear. *Neurosci. Biobehav. Rev.* 77 247–285. 10.1016/j.neubiorev.2017.02.026 28263758

[B44] Lundh L.-G. Öst L.-G. (1996). Recognition bias for critical faces in social phobics. *Behav. Res. Ther.* 34 787–794. 10.1016/0005-7967(96)00035-6 8952121

[B45] Macmillan N. A. Creelman C. D. (1991). *Detection Theory: A User’s Guide.* New York, NY: Psychology Press.

[B46] Mallan K. M. Sax J. Lipp O. V. (2009). Verbal instruction abolishes fear conditioned to racial out-group faces. *J. Exp. Soc. Psychol.* 45 1303–1307. 10.1016/j.jesp.2009.08.001 22091639

[B47] Mattick R. P. Clarke J. C. (1998). Development and validation of measures of social phobia scrutiny fear and social interaction anxiety. *Behav. Res. Ther.* 36 455–470. 10.1016/s0005-7967(97)10031-6 9670605

[B48] Mertens G. Boddez Y. Sevenster D. Engelhard I. M. De Houwer J. (2018). A review on the effects of verbal instructions in human fear conditioning: empirical findings, theoretical considerations, and future directions. *Biol. Psychol.* 137 49–64. 10.1016/j.biopsycho.2018.07.002 29990522

[B49] Milad M. R. Goldstein J. M. Orr S. P. Wedig M. M. Klibanski A. Pitman R. K. (2006). Fear conditioning and extinction: influence of sex and menstrual cycle in healthy humans. *Behav. Neurosci.* 120 1196–1203. 10.1037/0735-7044.120.5.1196 17201462

[B50] Mineka S. Oehlberg K. (2008). The relevance of recent developments in classical conditioning to understanding the etiology and maintenance of anxiety disorders. *Acta Psychol.* 127 567–580. 10.1016/j.actpsy.2007.11.007 18226795

[B51] Mühlberger A. Bülthoff H. H. Wiedemann G. Pauli P. (2007). Virtual reality for the psychophysiological assessment of phobic fear: responses during virtual tunnel driving. *Psychol. Assess.* 19 340–346. 10.1037/1040-3590.19.3.340 17845125

[B52] Mühlberger A. Weik A. Pauli P. Wiedemann G. (2006). One-session virtual reality exposure treatment for fear of flying: 1-year follow-up and graduation flight accompaniment effects. *Psychother. Res.* 16 26–40. 10.1080/10503300500090944 21827246

[B53] Mühlberger A. Wieser M. J. Pauli P. (2008). Visual attention during virtual social situations depends on social anxiety. *CyberPsychol. Behav.* 11 425–430. 10.1089/cpb.2007.0084 18721090

[B54] Neumann I. D. Slattery D. A. (2016). Oxytocin in general anxiety and social fear: a translational approach. *Biol. Psychiatry* 79 213–221. 10.1016/j.biopsych.2015.06.004 26208744

[B55] Pérez-López J. R. Woody S. R. (2001). Memory for facial expressions in social phobia. *Behav. Res. Ther.* 39 967–975. 10.1016/s0005-7967(00)00103-0 11480837

[B56] Pineles S. L. Orr M. R. Orr S. P. (2009). An alternative scoring method for skin conductance responding in a differential fear conditioning paradigm with a long-duration conditioned stimulus. *Psychophysiology* 46 984–995. 10.1111/j.1469-8986.2009.00852.x 19558401 PMC2868319

[B57] Powers M. B. Emmelkamp P. M. (2008). Virtual reality exposure therapy for anxiety disorders: a meta-analysis. *J. Anxiety Disord.* 22 561–569. 10.1016/j.janxdis.2007.04.006 17544252

[B58] Prescott L. Durkin M. Furchtgott E. Powell D. A. (1992). Concomitant heart rate and eyeblink pavlovian conditioning in human subjects as a function of interstimulus interval. *Psychophysiology* 29 646–656. 10.1111/j.1469-8986.1992.tb02040.x 1461955

[B59] Rapee R. M. Heimberg R. G. (1997). A cognitive-behavioral model of anxiety in social phobia. *Behav. Res. Ther.* 35 741–756. 10.1016/s0005-7967(97)00022-3 9256517

[B60] Reichenberger J. Porsch S. Wittmann J. Zimmermann V. Shiban Y. (2017). Social fear conditioning paradigm in virtual reality: social vs. electrical aversive conditioning. *Front. Psychol.* 8:1979. 10.3389/fpsyg.2017.01979 29250000 PMC5715328

[B61] Sawchuk C. N. Meunier S. A. Lohr J. M. Westendorf D. H. (2002). Fear, disgust, and information processingin specific phobia: the application of signal detection theory. *Anxiety Disord.* 16 495–510. 10.1016/s0887-6185(02)00168-8 12396208

[B62] Schubert T. W. (2003). Präsenzerleben in virtuellen umgebungen: eine skala zur messung von räumlicher präsenz, involviertheit und realitätsurteil. *Zeitschrift für Medienpsychologie* 15 69–71. 10.1026//1617-6383.15.2.69

[B63] Schwarz K. A. Wieser M. J. Gerdes A. B. Mühlberger A. Pauli P. (2013). Why are you looking like that? How the context influences evaluation and processing of human faces. *Soc. Cogn. Affect. Neurosci.* 8 438–445. 10.1093/scan/nss013 22287265 PMC3624952

[B64] Shiban Y. Fruth M. B. Pauli P. Kinateder M. Reichenberger J. Mühlberger A. (2016). Treatment effect on biases in size estimation in spider phobia. *Biol. Psychol.* 121 146–152. 10.1016/biopsycho.2016.03.005 26987423

[B65] Shiban Y. Pauli P. Mühlberger A. (2013). Effect of multiple context exposure on renewal in spider phobia. *Behav. Res. Ther.* 51 68–74. 10.1016/j.brat.2012.10.007 23261707

[B66] Shiban Y. Reichenberger J. Neumann I. D. Mühlberger A. (2015). Social conditioning and extinction paradigm: a translational study in virtual reality. *Front. Psychol.* 6:400. 10.3389/fpsyg.2015.00400 25904889 PMC4387857

[B67] Sosic Z. Gieler U. Stangier U. (2008). Screening for social phobia in medical in- and outpatients with the german version of the social phobia inventory (SPIN). *J. Anxiety Disord.* 22 849–859. 10.1016/j.janxdis.2007.08.011 17923381

[B68] Stangier U. Heidenreich T. Berardi A. Golbs U. Hoyer J. (1999). Die erfassung sozialer phobie durch die social interaction anxiety scale (SIAS) und die social phobia scale (SPS). *Zeitschrift Fur Klinische Psychologie* 28 28–36. 10.1026//0084-5345.28.1.28

[B69] Stangier U. Steffens M. (2002). *Social Phobia Inventory (SPIN) - Deutsche Fassung.* Frankfurt: Psychologisches Institut der Universität.

[B70] Stumpf H. Angleitner A. Wieck T. Jackson D. Beloch-Till H. (1985). *Deutsche Personality Research Form (PRF).* Boston, MA: Hogrefe.

[B71] Taylor S. Zvolensky M. J. Cox B. J. Deacon B. Heimberg R. G. Ledley D. R. (2007). Robust dimensions of anxiety sensitivity: development and initial validation of the anxiety sensitivity index-3. *Psychol. Assess.* 19 176–188. 10.1037/1040-3590.19.2.176 17563199

[B72] Velden M. (1982). *Die Signalentdeckungstheorie in der Psychologie.* Stuttgart: Kohlhammer.

[B73] Vervliet B. Geens M. (2014). Fear generalization in humans: impact of feature learning on conditioning and extinction. *Neurobiol. Learn. Mem.* 113 143–148. 10.1016/j.nlm.2013.10.002 24120427

[B74] Wieser M. J. Brosch T. (2012). Faces in context: a review and systematization of contextual influences on affective face processing. *Front. Psychol.* 3:471. 10.3389/fpsyg.2012.00471 23130011 PMC3487423

[B75] Wieser M. J. Pauli P. Alpers G. W. Mühlberger A. (2009). Is eye to eye contact really threatening and avoided in social anxiety? An eye-tracking and psychophysiology study. *J. Anxiety Disord.* 23 93–103. 10.1016/j.janxdis.2008.04.004 18534814

[B76] Zoicas I. Slattery D. A. Neumann I. D. (2014). Brain oxytocin in social fear conditioning and its extinction: involvement of the lateral septum. *Neuropsychopharmacology* 39 3027–3035. 10.1038/npp.2014.156 24964815 PMC4229574

[B77] Zorawski M. Blanding N. Q. Kuhn C. M. LaBar K. S. (2006). Effects of stress and sex on acquisition and consolidation of human fear conditioning. *Learn. Mem.* 13 441–450. 10.1101/lm.189106 16847304 PMC1538921

